# Post-translational regulation of autophagy is involved in intra-microbiome suppression of fungal pathogens

**DOI:** 10.1186/s40168-021-01077-y

**Published:** 2021-06-06

**Authors:** Jing Wang, Chaoyun Xu, Qiming Sun, Jinrong Xu, Yunrong Chai, Gabriele Berg, Tomislav Cernava, Zhonghua Ma, Yun Chen

**Affiliations:** 1 State Key Laboratory of Rice Biology, and Key Laboratory of Molecular Biology of Crop Pathogens and Insects, Institute of Biotechnology, Zhejiang University, 866 Yuhangtang Road, Hangzhou, 310058 China; 2 Department of Biochemistry, and Department of Cardiology of the Second Affiliated Hospital, Zhejiang University School of Medicine, Hangzhou, 310058 China; 3 Department of Botany and Plant Pathology, Purdue University, West Lafayette, IN USA; 4 Department of Biology, Northeastern University, Boston, MA USA; 5 Institute of Environmental Biotechnology, Graz University of Technology, Graz, Austria

**Keywords:** Intra-microbiome, Bacterial–fungal interaction, Autophagy, Post-translational regulation, Acetylation, *Fusarium graminearum*, *Streptomyces hygroscopicus*

## Abstract

**Background:**

Microbiome interactions are important determinants for ecosystem functioning, stability, and health. In previous studies, it was often observed that bacteria suppress potentially pathogenic fungal species that are part of the same plant microbiota; however, the underlying microbe-microbe interplay remains mostly elusive. Here, we explored antagonistic interactions of the fungus *Fusarium graminearum* and bacterium *Streptomyces hygroscopicus* at the molecular level. Both are ubiquitous members of the healthy wheat microbiota; under dysbiosis, the fungus causes devastating diseases.

**Results:**

In co-cultures, we found that *Streptomyces* alters the fungal acetylome leading to substantial induction of fungal autophagy*.* The bacterium secrets rapamycin to inactivate the target of rapamycin (TOR), which subsequently promotes the degradation of the fungal histone acetyltransferase Gcn5 through the 26S proteasome. Gcn5 negatively regulates fungal autophagy by acetylating the autophagy-related protein Atg8 at the lysine site K13 and blocking cellular relocalization of Atg8. Thus, degradation of Gcn5 triggered by rapamycin was found to reduce Atg8 acetylation, resulting in autophagy induction in *F. graminearum*.

**Conclusions:**

Autophagy homeostasis plays an essential role in fungal growth and competition, as well as for virulence. Our work reveals a novel post-translational regulation of autophagy initiated by a bacterial antibiotic. Rapamycin was shown to be a powerful modulator of bacteria–fungi interactions with potential importance in explaining microbial homeostasis in healthy plant microbiomes. The autophagic process provides novel possibilities and targets to biologically control pathogens.

**Video abstract**

**Supplementary Information:**

The online version contains supplementary material available at 10.1186/s40168-021-01077-y.

## Introduction

A balanced microbiome is important for human, plant, and environmental health, while diseases are often associated with microbial dysbiosis [1]. Dysbiotic microbiomes generally vary more in community composition than those from healthy individuals [2]. Despite numerous studies focusing on defining a healthy microbiome [3], only a few components and characteristics were identified so far [4]. Microbiome richness, evenness, and network complexity were repeatedly shown to be crucial for the health and balanced host–microbe interactions [5, 6]. However, detailed mechanisms that ensure balanced host–microbe interactions remain largely unclear. Recent findings indicate the specific importance of intra-microbiome interactions of bacteria and fungi [4, 7–10]. In plant microbiomes, bacteria and fungi often show negative co-occurrence trends in such communities, which indicates that the former suppress the latter [11]. This is important in connection with recent evidence that the microbiota of a healthy host can naturally harbor various pathogenic fungi, which can result in disease outbreaks when they are enriched [12]. While direct antagonism between bacteria and fungi based on antibiosis and response was often described, more complex interplay such as post-translation-mediated physiology and autophagy, is not well understood and thus not considered in current concepts for intra-microbiome interactions and modulations [13, 14].

Autophagy as a stress response plays a key role in the survival of eukaryotes [15, 16]. This is a highly conserved physiologic process; to date, 42 autophagy-related (Atg) proteins have been identified that are required for autophagic vacuole formation and development [17]. Among them, the ubiquitin-like protein Atg8 in yeast and its homolog LC3 in mammals are key regulators of autophagy that control major steps in the autophagic pathway and have been used in various studies as reliable markers for the induction and progression of autophagy [18]. Autophagy induction mainly depends on the serine/threonine protein kinase TOR (target of rapamycin) by phosphorylating core Atg proteins [19–21]. In yeast, TOR directly phosphorylates Atg13 causing a reduced affinity between Atg1 and subsequently inhibits the initiation of autophagy under nutrient-rich conditions [19, 22]. In mammals, mTORC1 (mammalian target of rapamycin complex 1) represses autophagy through the regulation of the ULK1 (a homolog of yeast Atg1)-Atg13-FIP200 protein complex formation by directly phosphorylating ULK1 at Ser758 under nutrient-rich conditions [23, 24]. Inactivation of mTORC1 results in auto-phosphorylation and upregulation of ULK1 kinase activity. In this case, ULK1 phosphorylates Atg13 and FIP200 and stimulates the ULK1-Atg13-FIP200 complex formation which initiates autophagy [23, 24]. In addition to phosphorylation of ULK1, mTORC1 was also shown to indirectly control autophagy through the phosphorylation of autophagy/Beclin-1 regulator 1 (AMBRA1), preventing ubiquitination of ULK1 to enhance its kinase activity in response to starvation or mTORC1 inhibition [20, 25]. Moreover, acetylation is also increasingly recognized as a post-translational modification for the regulation of autophagy [26]. Multiple Atg proteins have been shown to undergo changes in their acetylation statuses [27–31]. While it is known that both TOR induction and acetylation of Atg proteins are involved in controlling the autophagy process, their cross-regulation in autophagy remains less understood. Autophagy has been shown to play an important role in various cross-kingdom interactions, such as microbe-plant [32] and microbe-mammal [33, 34] interactions. However, targeted studies on the role of autophagy in microbe–microbe interactions are still limited [35].

The plant microbiome was identified as a key for the next green revolution (Science Breakthrough by 2030); however, we still need a better understanding of microbe–microbe interactions within the microbiota, especially related to antagonistic interactions between fungi and bacteria. *Fusarium graminearum* is the major causal agent of Fusarium head blight in wheat, which is a devastating fungal disease of wheat worldwide [36]. The pathogen is not only responsible for high yield losses in the field, it also produces harmful mycotoxins [36, 37]. The fungus belongs to the group of soil-borne pathogens, for which a dramatic increase is predicted due to intensification of agriculture, missing crop rotation, and climate change [38]. Due to missing plant resistance mechanisms, bacteria with antagonistic potential towards soil-borne fungi provide an alternative way to improve soil and plant health [14]. Here, members of the bacterial genus *Streptomyces*, which are known to produce a broad range of antimicrobial compounds and frequently coexist with *Fusarium* in natural niches [39, 40], are among the most promising candidates. Exploration of the underlying mechanisms of intra-microbiome interactions between *Streptomyces* and *Fusarium* will enhance our understanding of healthy microbiomes [40]. The objective of this study was to disentangle the interaction between two widespread components of the plant microbiota at the molecular level. Therefore, we isolated wheat-associated *Streptomyces* strains and subsequently focused on the most active isolate and potential biological control agent *S. hygroscopicus* S89. This isolate was able to induce fungal autophagy and alter the acetylome of *F. graminearum* in co-cultures. Rapamycin secreted by S89 was identified as the main effector in a potentially widespread intra-microbiome interaction.

## Methods

### *Streptomyces* spp. isolation, identification, and assessment of antifungal activity

In order to isolate *Streptomyces* spp., different wheat (*Triticum aestivum* L., Jimai 22) tissues (including spikelets, stem, leaves, and root), rhizosphere soil samples, and soil samples (soil samples were collected at a depth of 20 cm in wheat fields) were used. Isolation of bacteria was conducted by following established homogenization and soil dilution methods modified based on a previous study [41]. In brief, wheat tissues were ground thoroughly to complete homogeneity in a mortar with liquid nitrogen. The dry soil was mixed with 0.85% NaCl solution. Macerated samples were tenfold serially diluted and plated onto ISP2 agar plates supplemented with the fungicide Carbendazim to prevent the growth of fungi. *Streptomyces* colonies with characteristic morphology were further purified by sub-culturing. The pure isolates were preserved in sterilized glycerol (20%) as suspensions at − 20 °C. Subsequently, all *Streptomyces* spp. isolates were identified and classified based on their complete *16S rRNA* gene sequences. The sequence of each isolate was compared to reference sequences of other *Streptomyces* isolates deposited in NCBI’s nucleotide database with the BlastN algorithm. The antagonistic activity of *Streptomyces* isolates towards *F. graminearum* strain “PH-1” (NRRL 31084) was assessed in conventional dual-culture assay on CM plates. All isolates were tested in triplicates.

### Cultivation conditions for bacteria and fungi

For the acetylome analysis, 24-h complete medium (CM) culture broths of *Streptomyces hygroscopicus* S89 as well as its culture supernatants were mixed with equal volumes of CM broth containing 12-h cultures of *F. graminearum* and co-incubated for 0–12 h. For the identification of active compounds of S89 and the model strain NRRL5491, the *Streptomyces* strains were grown in CM broth for 5 days at 30 °C in a shaker and their mycelia were subsequently collected by centrifugation (10,000*g*, 10 min). Then an equal volume of ethanol was added and the suspension was incubated at 50 °C for 180 min with repeated vortexing after every 15 min. Subsequently, another centrifugation (10,000*g*, 10 min) was performed to remove the precipitated cell mass; the supernatant was filtered and subjected to LC-MS (liquid chromatography-mass spectrometry) analyses. Detailed components of different media were provided in the supplementary Materials and Methods.

### Autophagy experiments

Mutant strains were generated from the GFP-Atg8 labeled *F. graminearum* PH-1 strain. For cleavage analyses of GFP-Atg8 via western blot, mycelia of each strain were cultivated in liquid complete medium (CM) for 12 h, then transferred into nitrogen-free minimal medium (MM-N) or treated with 25 nM rapamycin for 0–8 h before harvest. For microscopy observations, mycelia that were starved or cultivated under nutrient-rich conditions or/and treated with rapamycin (25 nM), MG132 (50 μM), and 3-MA (100 μM) in the presence/absence of bafilomycin A1 (2 μM) were placed on glass slides and visualized with a Zeiss LSM780 confocal microscope (Gottingen, Niedersachsen, Germany). They were stained with CMAC (CellTracker™ Blue CMAC (7-amino-4-chloromethylcoumarin), Invitrogen, C2110) or DAPI (4′, 6-diamidino-2-phenylindole, Invitrogen, D1306) to mark vacuoles and the nucleus respectively. For transmission electron microscopy (TEM), CM-cultivated mycelia were transferred into MM-N for 4 h in the presence of 4 mM phenylmethanesulfonyl fluoride (PMSF). Mycelia of different *F. graminearum* strains that were obtained with the abovementioned cultivation conditions were harvested and fixed with 2.5% glutaraldehyde phosphate buffer, dehydrated with 1% osmium tetroxide cacodylate buffer and a sequential ethanol gradient, and then immersed in epoxy resin afterwards. Ultrathin sections were placed on carbon-coated copper grids and counterstained with uranyl acetate and lead citrate. Images were taken with a transmission electron microscope (JEM-1230, JEOL, Japan).

### Immunoblot assays and immunoprecipitation

For immunoblot analysis of the acetylome, autophagy flux, and the concentration of Gcn5, freshly harvested mycelia of *F. graminearum* were lysed in 8 M urea buffer supplemented with a 1 mM complete protease inhibitor cocktail (Sangon biotech (Shanghai) Co., Ltd., China). Proteins were resolved on SDS polyacrylamide gels and then transferred to a polyvinylidene difluoride membrane. After blocking with 5% (w/v) bovine serum albumin, the membrane was incubated sequentially with the corresponding primary and secondary antibodies. Information related to the antibodies used in this study is provided in the supplementary Materials and Methods. Each experiment was conducted independently three times at least. All blots were imaged with the ImageQuant LAS 4000 mini (GE Healthcare, Chicago, USA). The specific bands were quantified using ImageJ software (v1.8.0, National Institutes of Health).

For immunoprecipitation, 200 mg freshly freeze-dried mycelia of each strain was lysed in 1 mL extraction buffer (50 mM Tris-HCl, pH 7.5, 100 mM NaCl, 5 mM EDTA, 1% Triton X-100, 2 mM PMSF) supplemented with a complete protease inhibitor cocktail. Then immunoprecipitation was performed using the aforementioned antibodies before the addition of protein A/G agarose or using corresponding affinity agarose directly. Briefly, 2 μg of the antibody was added to 1 mL of mycelia lysate and incubated for 6 h at 4 °C. After the addition of protein A/G agarose beads, incubation was continued for 2 h at 4 °C. For direct use of antibody-coupled agarose, incubation only requires 6 h. Immunoprecipitation complexes were washed six times with washing buffer (50 mM Tris-HCl, pH 8.0, 150 mM NaCl, 1% NP40), resolved by SDS-PAGE, and analyzed via western blot (further information is provided in the supplementary Materials and Methods).

### Quantification and statistical analysis

In vitro experiments and in vivo assay were repeated at least three times. The extent of autophagy was estimated by calculating the proportion of free GFP compared to the total amount of intact GFP-Atg8 plus free GFP. The respective band intensities were quantified with ImageJ 1.8.0. All statistical analyses were performed using SAS software (V9.2, SAS Institute INC). Data were presented as mean ± standard deviation (s. d.). Differences between two groups were assessed by using a two-tailed Student’s t test. Multiple comparisons were assessed by one-way analysis of variance (ANOVA) followed by a least significant difference (LSD) multiple-range test.

## Results

### *S. hygroscopicus* S89 induces autophagy in *F. graminearum*

**Fig. 1:**
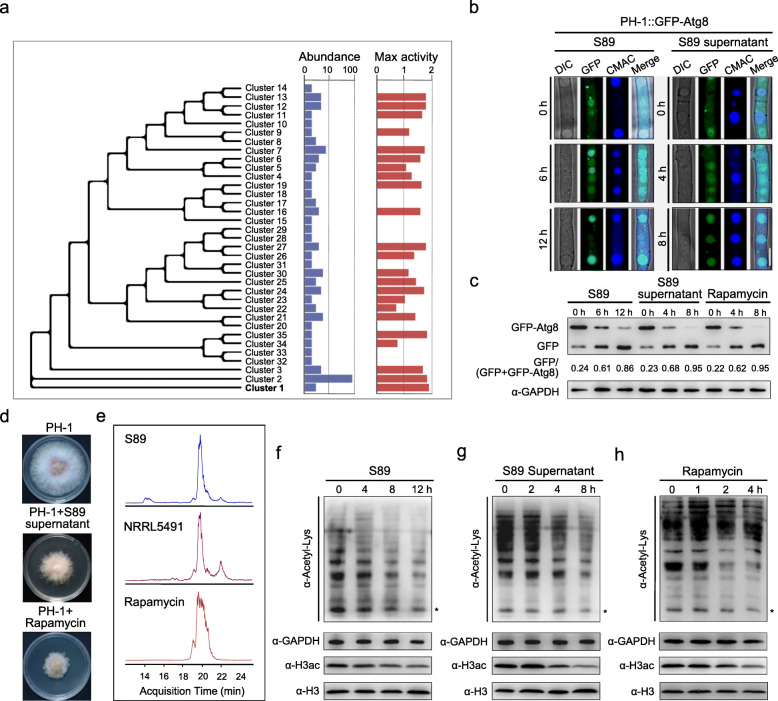
Rapamycin secreted by *Streptomyces hygroscopicus* S89 alters fungal autophagy and global acetylome in *Fusarium graminearum*. **a** Phylogenetic tree of *Streptomyces* strains. Bar plots on the right describe the abundance (number) and the radius of antagonistic inhibition zone (cm) of tested bacterial strains. **b** Fungal autophagy was induced by S89 and its supernatant. The wild-type *F. graminearum* strain PH-1 labeled with GFP-Atg8 was co-cultured with S89 or supernatant, and the GFP-Atg8 translocation in mycelia was observed using a confocal microscope. Vacuoles were labeled with the CMAC dye. Bar = 10 μm. **c** Fungal autophagy flux in the strain PH-1::GFP-Atg8 upon various treatments. Total proteins extracted from mycelia were immunoblotted using the anti-GFP antibody. The extent of autophagy was estimated by calculating the amount of free GFP vs the total amount of intact GFP-Atg8 plus free GFP (the numbers appear underneath the blot). The protein samples were also detected with anti-GAPDH antibody as a loading control. **d** Antifungal activity of S89 supernatant and rapamycin. **e** LC-MS profile of rapamycin purified from the supernatant of S89 and the model strain *S. hygroscopicus* NRRL5491. Pure rapamycin was used as a positive control. **f**–**h** Acetylome profiles of *F. graminearum* in co-cultures with S89, S89 supernatant, or rapamycin. Total proteins were immunoblotted using the indicated antibodies. The anti-acetylated-lysine antibody (α-Acetyl-Lys) was used for detecting acetylated proteins, and the anti-acetyl-histone H3 antibody (α-H3ac) for detecting histone 3 acetylation. Proteins samples were also detected with anti-GAPDH or anti-H3 antibody as a loading control. Asterisks indicate histone bands

A total of 180 *Streptomyces* strains was isolated from wheat tissues and the rhizosphere. Phylogenetic analysis indicated that these isolates are genetically diverse and can be distributed into 35 clusters, based on their 16S *rRNA* gene sequences (> 99% identity) (Fig. 1a). A high proportion of the strains (70%) demonstrated a varying degree of inhibitory activity against mycelial growth of *F. graminearum* strain PH-1 (NRRL 31084) in vitro (Table S1). Moreover, there was a high correlation between *Streptomyces* phylogeny and their strength of inhibition against fungal growth (Pagel’s λ value = 0.63, *P* = 0.001), which implies that certain *Streptomyces* species were better adapted to counteract *F. graminearum* in their natural environment.

Notably, isolate S89 demonstrated the highest inhibitory activity, producing radial of inhibition zones > 18 mm on complete medium (CM). S89 was identified as *S. hygroscopicus* based on its complete 16S *rRNA* gene sequence (99.93% identity). More interestingly, when the fungus was co-cultured with *S. hygroscopicus* S89 in liquid CM, we observed that the GFP-Atg8 fusion protein, an autophagy flux indicator in *F. graminearum*, continuously translocated into vacuoles, compared with that in pure fungal cultures (Fig. 1b, S1a). Immunoblot assays further showed that the amount of cleaved GFP (cleaved from GFP-Atg8 fusion proteins when autophagosomes fused with vacuoles) increased with increasing co-cultivation times (Fig. 1c). The observation indicated that the autophagy flux in *F. graminearum* was substantially increased during co-cultivation. These findings prompted us to explore the mechanism of autophagy induction in this fungus by S89, and its role in this antagonistic cross-kingdom interaction.

Antagonistic interactions between members of *Streptomyces* and *Fusarium* are known to be largely mediated by antibiotics produced by *Streptomyces* [40]. To evaluate this, we first tested and confirmed that the S89 supernatant can suppress fungal growth and induce autophagy (Fig. 1b–d). The active antimicrobial compounds that are secreted by S89 were purified and their subsequent identification showed that rapamycin was one of the actives in the S89 supernatant (Fig. 1e, S1b). Rapamycin is a natural compound that was previously found in *S. hygroscopicus* [42]. We found that rapamycin significantly stimulated autophagy and inhibited mycelial growth in *F. graminearum* (Fig. 1c, d). Taken together, rapamycin secreted by S89 was found to be the main effector in bacterial–fungal interactions (BFI) between *S. hygroscopicus* S89 and *F. graminearum*.

### Inactivation of TOR reduces the global acetylome in *F. graminearum*

During the co-cultivation of *S. hygroscopicus* S89 and *F. graminearum*, global acetylation of histones and non-histone proteins in *F. graminearum* was found to be significantly reduced. This was also observed upon treatment with S89 supernatant and pure rapamycin (Fig. 1f–h). In particular, the level of histone 3 acetylation (H3ac) in the wild type *F. graminearum* PH-1 was significantly decreased upon treatment (Fig. 1f–h). These results suggested that rapamycin-mediated TOR inhibition may reduce acetyltransferase activities, especially histone acetyltransferase(s) in this fungus.

**Fig. 2:**
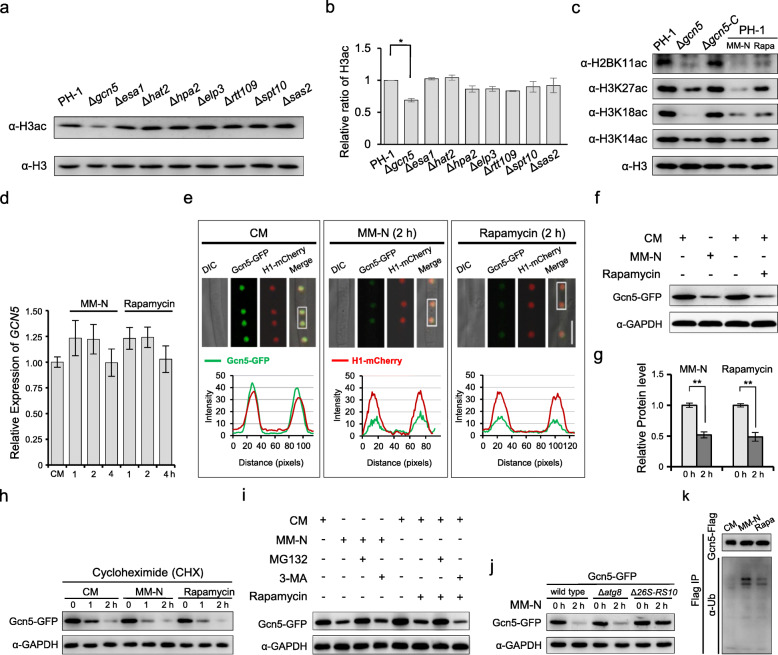
Inhibition of TOR reduces the acetylome through Gcn5. **a** The level of H3ac in histone acetyltransferases (HATs) disruption mutants. **b** Quantification of the relative levels of H3ac/H3 in (**a**). **c** Effects of *GCN5* deletion, rapamycin (Rapa) treatment, and nitrogen starvation (MM-N) on histone acetylation. Total proteins extracted from mycelia of tested strains with or without treatment were immunoblotted using indicated antibodies. **d** Transcription of *GCN5* detected by RT-PCR in the mycelia of PH-1 grown in completed medium (CM) and under autophagy induction condition (MM-N). **e** CM and rapamycin/nitrogen starvation–treated Gcn5-GFP-expressing mycelia were observed with a confocal microscope; intensity of Gcn5-GFP was quantified with ImageJ. Bar = 10 μm. **f**, **g** Relative amount of Gcn5-GFP under tested conditions in (**e**). Samples were analyzed using immunoblot assays. Data are presented as mean ± s.d from triplicates. Asterisks indicate significant difference according to a LSD test at *P*< 0.01. **h** Relative amount of Gcn5-GFP under CM, MM-N, or CM with rapamycin (25 nM) in the presence of the translational inhibitor, cycloheximide (CHX). **i** MG132 or 3-MA was added into nitrogen-free minimal or CM with (out) rapamycin medium for 2 h, then mycelia were harvested and lysed and the degradation of Gcn5-GFP was detected with immunoblotting. **j** Degradation of Gcn5-GFP in the 26S proteasome–defective mutant Δ*26S-RS10*, or the autophagy of defective mutant Δ*atg8*. **k** The ubiquitination level of Gcn5-Flag under autophagy induction conditions

To identify the TOR-related histone acetyltransferases (HATs), we analyzed the role of 8 putative HATs [14] on histone 3 acetylation (H3ac) by immunoblotting in *F. graminearum*. As shown in Fig. 2a, b, deletion of *GCN5*, but not other HAT genes, resulted in a noticeable reduction of the H3ac levels. Furthermore, inactivation of TOR by rapamycin reduced acetylation levels of histones H2BK11, H3K14, H3K18, and H3K27, similar to what was observed with the *F. graminearum GCN5* deletion mutant (Fig. 2c). Nitrogen starvation is known as another approach to inactivate TOR [43]. We also found that nitrogen starvation (by using minimal medium without nitrogen, MM-N) triggered the autophagy flux and reduced the acetylome, similar to the treatment with rapamycin or S89 supernatant (Fig. 2c, S1c-d). Taken together, inactivation of TOR by rapamycin or nitrogen starvation resulted in a reduced global acetylome in *F. graminearum*, and this is likely mediated by reduced HAT activities of Gcn5.

### TOR inhibition promotes the degradation of the acetyltransferase Gcn5 through 26S proteasome

We next explored how TOR regulates the activity of Gcn5. Therefore, we first examined the transcriptional level of *GCN5* during autophagy induced by rapamycin or nitrogen starvation using qRT-PCR. As shown in Fig. 2d, there was no significant change in the transcription of *GCN5* under autophagy induction, compared with non-induction. Next, we monitored the Gcn5 protein level and cellular localization of Gcn5 by analyzing the fluorescent intensity of the Gcn5-GFP construct in the mycelia of a dual-labeled wild-type strain expressing both Gcn5-GFP and histone 1 (H1)-mCherry. Gcn5-GFP was co-localized with H1-mCherry in the nucleus of mycelia grown in CM (Fig. 2e). Interestingly, the relative fluorescent intensity of Gcn5-GFP was substantially decreased during autophagy induced by nitrogen starvation (MM-N) and rapamycin (Fig. 2e). The changes in the Gcn5-GFP protein level under different conditions were further verified by immunoblots using an anti-GFP antibody. In agreement with the microscopic observation, the level of Gcn5-GFP was significantly reduced under autophagy induction (Fig. 2f, g). To test whether the decreased level of Gcn5-GFP was caused by an inefficient translation, mycelia of the Gcn5-GFP labeled strain were treated with the translational inhibitor cycloheximide (CHX). The protein level of Gcn5-GFP in the mycelia treated with the CHX under autophagy-inducing conditions was less than that in the mycelia treated only with CHX (Fig. 2h). These results indicate that Gcn5 is likely degraded under autophagy-inducing conditions.

The ubiquitin–proteasome system and the autophagy-lysosome system represent two main protein degradation pathways in eukaryotic cells [44]. To explore which degradation pathway controls Gcn5 turnover during autophagy induction, a 26S proteasome inhibitor [z-Leu-Leu-Leu-CHO (MG132)] and an autophagy inhibitor 3-methyladenine (3-MA) were added to the fungal culture in either MM-N or CM supplemented with rapamycin. Treatments with MG132, but not 3-MA, restored Gcn5 protein levels under both nitrogen starvation (MM-N) and in the rapamycin treatment (Fig. 2i), although 3-MA is able to effectively suppress the autophagy process in *F. graminearum* (Figure S2). These results suggested that Gcn5 protein degradation is likely mediated by the 26S proteasome during autophagy.

To further confirm that the 26S proteasome is involved in the Gcn5 degradation during autophagy induction we constructed autophagy and 26S proteasome pathway deficient mutants by disrupting the *ATG8* and 26S proteasome regulatory subunit 10 (designated as *26S-RS10*, locus FGSG_01338), respectively, in the Gcn5-GFP complementation strain. Subsequently, Gcn5-GFP protein levels were determined in those strains. As shown in Fig. 2j, in the deletion mutant Δ*26S-RS10*, degradation of Gcn5-GFP was notably attenuated in comparison to that in the wild type, while the Δ*atg8* mutant had no clear influence on Gcn5-GFP degradation under the same condition. Furthermore, we examined the ubiquitination level of Flag-tagged Gcn5 with an immunoblot using an anti-ubiquitination monoclonal antibody, under rapamycin, nitrogen starvation, and nutrient-rich conditions. As expected, both rapamycin treatment and nitrogen starvation increased the ubiquitination level of Gcn5-Flag, but not under nutrient-rich conditions (Fig. 2k). Collectively, these results indicate that inactivation of TOR by rapamycin or nitrogen starvation promotes the degradation of Gcn5 and that Gcn5 degradation is mediated by the 26S proteasome.

### Gcn5 negatively regulates autophagy

To determine if Gcn5 degradation and the reduced acetylome are linked to autophagy induction, we generated a Δ*gcn5* deletion mutant as well as an in locus over-expression strain OE-*GCN5* in which the expression of *GCN5* is under the control of the *gpda* promoter from *Aspergillus nidulans* in the GFP-Atg8 labeled PH-1 (Figure S3a). The Δ*gcn5* mutant and OE-*GCN5* strain were verified by PCR amplification of the entire open reading frame of *GCN5* (Figure S3b). The transcriptional level of *GCN5* in OE-*GCN5* was also examined by qRT-PCR. It turned out to be 20-fold higher than that in the parent GFP-Atg8 strain (Figure S3c).

**Fig. 3:**
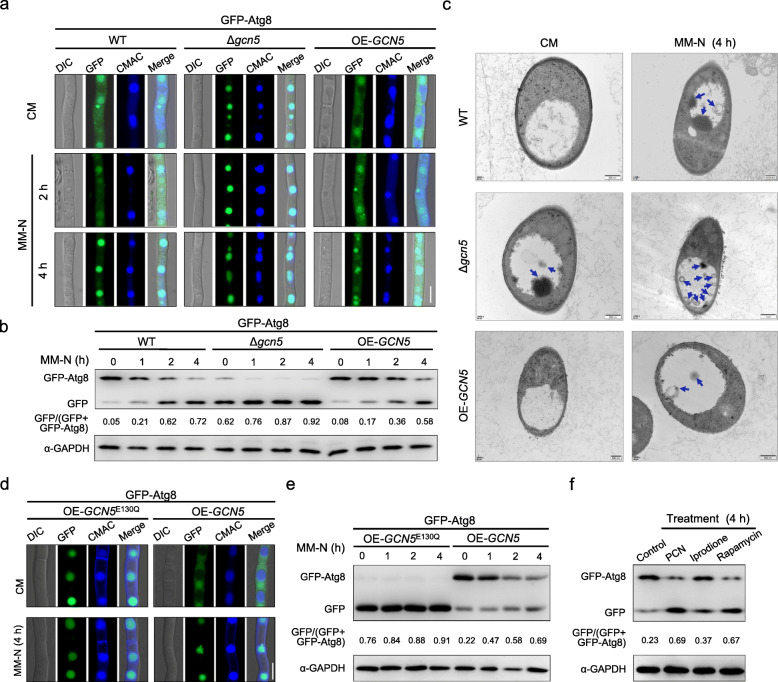
Gcn5 is a repressor of autophagy. **a** Confocal microscopy observation on GFP-Atg8 relocalization in mycelia of the wild type, Δ*gcn5*, and the over-expression strain OE-*GCN5*. Vacuoles were labeled with the CMAC dye. Bar = 10 μm. **b** Autophagy flux of the wild type (WT), Δ*gcn5*, and OE-*GCN5* under starvation conditions. **c** Transmission electron microscopy images of autophagic structures (blue arrows) in mycelia of WT, Δ*gcn5*, and OE-*GCN5* grown in CM or MM-N for 4 h. **d** Confocal microscopy observation on GFP-Atg8 relocalization in mycelia of OE-*GCN5* or the acetyltransferase enzymatic inactive mutant OE-*GCN5*^E130Q^. Mycelia were grown in CM or MM-N for 4 h. Bar = 10 μm. **e** Autophagy flux of Gcn5^WT^ and Gcn5^E130Q^ strain under starvation conditions. **f** Autophagy flux of the wild-type strain expressing GFP-Atg8 upon the enzymatic activity inhibitor of Gcn5, phenazine-1-carboxamide (PCN). Rapamycin was used as a positive control for autophagy induction, and the fungicide iprodione was used as a control to exclude the growth inhibition

We then examined the autophagy flux in the wild type, as well as the Δ*gcn5* and OE-*GCN5* constructs. Given that autophagy flux induced by nitrogen starvation is similar to that by rapamycin, we conducted subsequent autophagy experiments using MM-N. In the wild type strain, under nutrient-rich conditions (CM), the GFP-Atg8 fusion protein was detected as diffused fluorescent signal in the cytoplasm, mainly accumulated in spherical structures, but not in the vacuole, which can be visualized with Cell Tracker Blue CMAC (vacuole-specific molecular probe). Upon TOR inactivation by starvation, the GFP-Atg8 proteins translocated into the vacuoles, evidenced by CMAC staining and GFP signals after a 4-h induction (Fig. 3a, left panel). Interestingly, the starvation-induced GFP-Atg8 translocation to vacuoles was strongly promoted in the Δ*gcn5* mutant, where GFP signals had already been localized in vacuoles even before starvation (Fig. 3a, middle panel). On the contrary, over-expression of *GCN5* (OE-*GCN5*) impaired GFP-Atg8 translocation into vacuoles under starvation. A portion of GFP signals still showed a punctate pattern or remained in the cytoplasm even after a 4-h induction (Fig. 3a, right panel).

The autophagy level was further quantitatively analyzed by calculating the amount of free GFP versus the total amount of intact GFP-Atg8 plus free GFP via immunoblots [45]. In Δ*gcn5*, the autophagy level was significantly elevated as evidenced by the proportion of free GFP that was higher in the mutant than the wild-type at each tested time point. In contrast, in the OE-*GCN5* strain, autophagy activity was reduced (Fig. 3b). Furthermore, ultrastructural autophagic structures (autophagosome and autophagic body) were examined by electron microscopy. Under nutrient-rich conditions (CM), no noticeable autophagic structures were observed in the wild-type mycelia. After starvation, multilamellar-cupped membrane or membrane-surrounded autophagic structures were observed in cells (indicated with blue arrows) (Fig. 3c, upper panel). However, in the mycelia of Δ*gcn5* mutant, a few autophagic structures in the vacuole lumina were detected even before starvation and were further intensified upon starvation (Fig. 3c, middle panel). Conversely, formation and accumulation of autophagic structures were clearly decreased in OE-*GCN5* (Fig. 3c, bottom panel). Thus, results from ultrastructural analyses are in agreement with those from live-cell fluorescent microscopy and immunoblot analysis. Moreover, autophagy fluxes induced by rapamycin are similar to those induced by starvation in all tested strains (Figure S3d). Hence, our results suggested that Gcn5 is a negative regulator of autophagy.

To determine whether the negative regulation of autophagy by Gcn5 depends on its enzymatic activity, we constructed another in locus over-expression strain expressing an enzymatically inactive mutant of Gcn5, OE-*GCN5*^E130Q^ in the GFP-Atg8 background and analyzed the autophagy flux. The E130 residue (glutamic acid) is highly conserved in fungal Gcn5 homologs (Figure S4a) and critical for its HAT activity [46]. Amino acid substitution from E to Q (glutamine) would dismiss the HAT activity of Gcn5 [46]. As indicated by the results shown in Fig. 3d, e, the autophagy flux in OE-*GCN5*^E130Q^ strain was substantially promoted, while being very different from the overexpression of wild type *GCN5*, but similar to that in the Δ*gcn5* mutant. Phenazine-1-carboxamide (PCN) was identified as an inhibitor on the enzymatic activity of Gcn5 in our previous study [14]. The autophagy process was also elevated in the wild-type strain treated with PCN (Fig. 3f). Consistently, the HAT activity of Gcn5 was also critical for rapamycin-induced autophagy. Compared to the wild type, the number of GFP-Atg8 puncta in Δ*gcn5* and OE-*GCN5*^E130Q^ was significantly increased, while the OE-*GCN5* strain showed a decreased number of GFP-Atg8 puncta, in the presence of the vacuolar inhibitor bafilomycin A1 (Figure S4b-c). Taken all observations together, our results demonstrate that Gcn5 negatively regulates autophagy, and the regulation is largely dependent on its HAT activity. We hypothesize that upon rapamycin treatment or under nitrogen starvation, TOR inhibition triggers degradation of Gcn5, which in turn accelerates the autophagy flux in *F. graminearum*.

### Gcn5 acetylates the autophagy-related protein Atg8

**Fig. 4:**
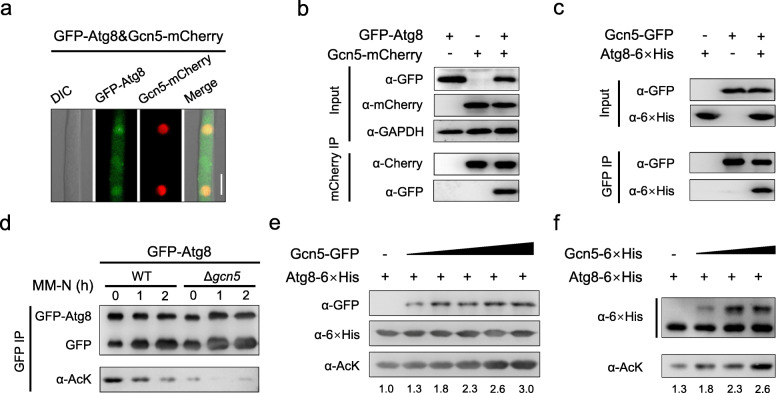
Atg8 is acetylated by Gcn5. **a** GFP-Atg8 and Gcn5-mCherry co-localized in nuclei. The dual-labeled strain was grown in CM and observed with a confocal microscope. Bar = 10 μm. **b** Co-IP analysis of the interaction between Atg8 and Gcn5. GFP-Atg8 and Gcn5-mCherry were detected using an anti-GFP or anti-mCherry antibody, respectively. Proteins samples were also detected with anti-GAPDH antibody as an internal control. **c** Pull-down analysis of Atg8-6×His and Gcn5-GFP. 6×His-tagged Atg8 purified from *E. coli* was incubated with the lysate of the fungal strain expressing Gcn5-GFP. Gcn5-GFP was immunoprecipitated using an anti-GFP antibody, and the precipitated complex was analyzed by immunoblotting using anti-His or anti-GFP antibody. **d** Acetylation level of Atg8 in WT or Δ*gcn5*. The GFP-Atg8 was immunoprecipitated with GFP-agarose from the whole mycelial lysate of the WT or Δ*gcn5* grown in CM or MM-N, the acetylation level of Atg8 was detected with an anti-pan-acetyl-lysine monoclonal antibody (α-AcK). **e**, **f** Acetylation of Atg8 catalyzed by Gcn5 in vitro. Purified Atg8-6×His from *E. coli* was incubated with increased amounts of Gcn5-GFP purified from *F. graminearum* (**e**) or Gcn5-6×His purified from *E. coli* (**f**) and then detected with α-AcK antibody. The relative intensities of acetylated Atg8 bands were quantified with ImageJ. The fold change is relative to the control reaction without Gcn5-GFP or Gcn5-6×His supplementation. The bands in the control reaction were set as 1.00

Gcn5 regulates numerous cellular processes through gene transcription or protein acetylation [47]. We speculated that Gcn5 likely plays roles in regulating the transcription of 26 *ATG* genes or Atg protein acetylation in *F. graminearum*. To test this hypothesis, we first compared the transcription of all 26 *ATG* genes in the wild type and the Δ*gcn5* mutant under nitrogen starvation by RNA-seq. Our data suggested no significant difference in the expression of most *ATG* genes in these two strains (Table S2). It thus ruled out the possibility that Gcn5 regulates autophagy via altering *ATG* gene transcription. We next tested whether Gcn5 influences autophagy by controlling acetylation of Atg proteins or other autophagy regulation proteins. Affinity capture mass spectrometry (ACMS) was used to identify Gcn5 interacting proteins using in locus over-expressed Gcn5-GFP as the bait. In the ACMS assay, Gcn5 and its well-known substrates histones H2B and H3, were all identified, which validated the reliability of the ACMS results. Notably, several proteins with known functions in autophagy regulation, including Atg8, were identified (Table S3). To further verify whether Gcn5 indeed interacts with Atg8, a dual-labeled strain with Gcn5-mCherry and GFP-Atg8 was constructed. As shown in Fig. 4a, GFP-Atg8 co-localized with Gcn5-mCherry in the nucleus of mycelia grown in CM, although some GFP-Atg8 signals were also present in the cytoplasm. The direct protein–protein interaction between Gcn5 and Atg8 was further confirmed by carrying out co-immunoprecipitation (Co-IP) and in vitro pull-down assays (Fig. 4b, c). In summary, our results from both in vivo and in vitro assays strongly suggest that Gcn5 directly interacts with Atg8.

The abovementioned findings prompted us to speculate that Atg8 is an important acetylation target of Gcn5 during autophagy. To test this hypothesis, the acetylation level of Atg8 in the wild type and the Δ*gcn5* mutant was compared. GFP-Atg8 proteins were purified from either the wild type or Δ*gcn5* mycelia grown in MM-N at different time points and analyzed by immunoblots with an anti-pan-acetyl-lysine monoclonal antibody (α-AcK). Acetylation of Atg8 was detected in both strains; however, the acetylation level was significantly reduced in the Δ*gcn5* mutant compared to the wild type (Fig. 4d). The acetylation level of Atg8 was also reduced significantly over time in the wild-type strain during autophagy induction by nitrogen starvation (MM-N, Fig. 4d). The decreased Atg8 acetylation could result from the degradation of Gcn5 during autophagy induction as we showed above (Fig. 2). Residual Atg8 acetylation levels detected in the Δ*gcn5* mutant, suggested that there are other acetyltransferase(s) targeting Atg8. We additionally performed Atg8 acetylation by Gcn5 in vitro. As shown in Fig. 4e, f, Atg8-6×His was partially acetylated when expressed and purified in *E. coli*. Importantly, the addition of an increasing amount of Gcn5 purified from either *F. graminearum* (Gcn5-GFP, Fig. 4e) or *E. coli* (Gcn5-6×His, Fig. 4f) elevated the acetylation level of Atg8 proportionally. We thus conclude that Atg8 is a direct acetylation substrate of Gcn5.

### K13 is the main acetylation site in Atg8 catalyzed by Gcn5

**Fig. 5:**
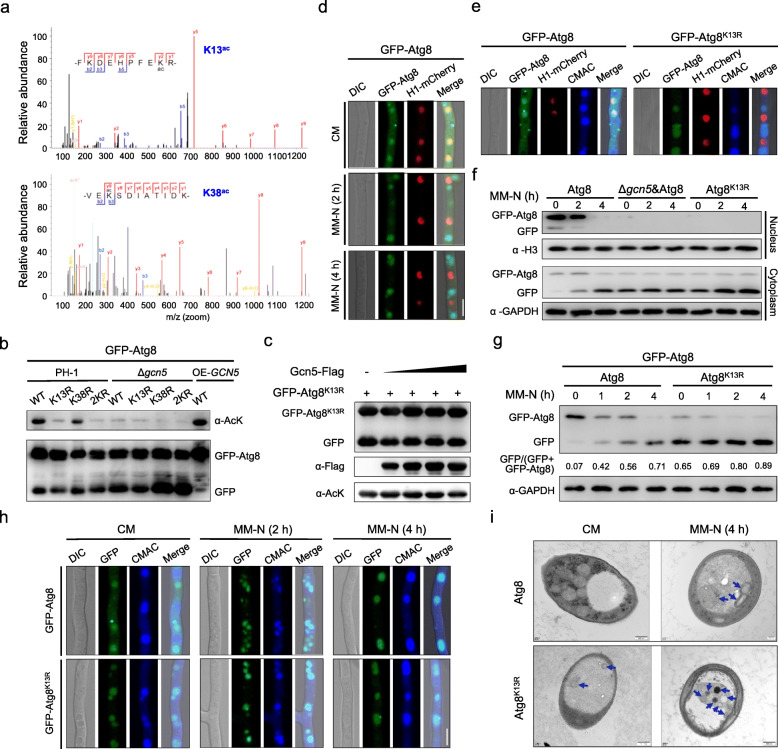
Gcn5 acetylates Atg8 at K13 and represses autophagy. **a** Mass spectrometry of the peptides containing K13 or K38 acetylation. **b** Acetylation level of GFP-tagged Atg8 or mutated Atg8 proteins. The double mutated Atg8^K13R-K38R^ was abbreviated to Atg8^2KR^. GFP tagged proteins were immunoprecipitated with GFP-agarose and immunoblotted by α-AcK. **c** In vitro acetylation assay using immunoprecipitated Atg8^K13R^ and Gcn5-Flag. **d** Translocation of GFP-Atg8 during autophagy process. Mycelia expressing GFP-Atg8 were grown in CM and then transferred into MM-N. The localization of GFP-Atg8 was observed at indicated time points by confocal microscopy. **e** Localization of the wild type GFP-Atg8 and Atg8^K13R^. Histone 1 (H1)-mCherry and CMAC staining served as nuclear and vacuolar markers respectively. Bar = 10 μm. **f** Protein abundance of GFP-Atg8 and GFP-Atg8^K13R^ in cytoplasm and nucleus. Cytoplasmic and nuclear GFP-Atg8 or GFP-Atg8^K13R^ were fractionated and analyzed by immunoblot assay using the anti-GFP antibody. GAPDH and Histone 3 (H3) were detected as internal controls for the cytoplasmic and nuclear fractions, respectively. **g** Autophagy flux of GFP-Atg8 and GFP-Atg8^K13R^ strain upon nitrogen starvation. **h** Relocalization of GFP-Atg8 or GFP-Atg8^K13R^ during autophagy induction. Bar = 10 μm. **i** TEM images of autophagic structures (blue arrows) in mycelia of GFP-Atg8 and GFP-Atg8^K13R^

To determine putative acetylation site(s) in Atg8 catalyzed by Gcn5, we purified the GFP-Atg8 fusion proteins from the mycelia of the wild type and the Δ*gcn5* mutant in nutrient-rich medium and analyzed sites of lysine acetylation using mass spectrometry. We repeatedly identified two acetylated lysine sites, K13 and K38, in Atg8 purified from the wild type but not from the Δ*gcn5* mutant (Fig. 5a). K13 and K38 are evolutionarily conserved across Atg8 homologs from fungi to insects (Figure S5a). To confirm these two lysine acetylation sites, we mutated K13 and/or K38 in Atg8 to R (arginine) and determined the acetylation level of mutated Atg8 proteins. Both Atg8^K13R^ and Atg8^K38R^ as well as the double-mutated Atg8^K13R-K38R^ (Atg8^2KR^) demonstrated significantly reduced acetylation (Fig. 5b, left panel), confirming that Atg8 can be acetylated at the K13 and K38 sites. It was also evident that the K13 site plays a more prominent role than K38 in Atg8 acetylation, since the Atg8^K13R^ transformant had an even lower acetylation level than Atg8^K38R^ (Fig. 5b).

The acetylation level of both the wild type and the mutated Atg8 showed a significant but similar reduction in the Δ*gcn5* background. In the *GCN5* over-expression strain, the acetylation level of the wild type Atg8 was notably enhanced, but the double-mutated Atg8^2KR^ still indicated a severe reduction of the acetylation level (Fig. 5b). Interestingly, K13 but not K38 acetylation was absent in the Δ*gcn5* mutant in the mass spectrometry assay, although the peptide containing this residue was covered. This indicates that acetylation at K13 in Atg8 might be specifically catalyzed by Gcn5. Furthermore, the acetylation level of Atg8^K13R^ purified from *F. graminearum* was not increased with increasing amounts of Gcn5 in vitro (Fig. 5c), in comparison with that of the wild type Atg8 (Fig. 4e, f). These results thus suggested that K13 in Atg8 is acetylated specifically by Gcn5.

### K13 acetylation in Atg8 suppresses the autophagy flux

Dynamic subcellular localization of GFP-Atg8 was observed during the autophagic process in *F. graminearum* upon TOR inactivation. GFP-Atg8 was mainly localized in the nucleus and overlapped with an H1-mCherry in nutrient-rich medium (CM) (Fig. 5d, upper panel). Upon nitrogen starvation, GFP-Atg8 moved out of the nucleus, relocalized to the cytoplasm and vesicle-like organelles (Fig. 5d, middle panel), and primarily resided in the vacuoles for longer time periods (Fig. 5d, bottom panel). Strikingly, the GFP-Atg8^K13R^ mutant was exported from the nucleus and localized in CMAC-stained vacuoles in most cells, even when cultured in CM (Fig. 5e). Moreover, deletion of *GCN5* reduced K13 acetylation in Atg8 and accelerated its relocalization to the cytoplasm, whereas over-expression of *GCN5* retained the localization of GFP-Atg8 in the nucleus (Figs. 3a and 5b). On the other hand, the GFP-Atg8^K38R^ mutant protein was still localized in the nucleus and did not change the autophagy flux (Figure S5b-c). Collectively, our results suggest that K13 acetylation in Atg8 catalyzed by Gcn5 is critical for its subcellular relocalization.

We next examined whether redistribution of GFP-Atg8^K13R^ participates in the autophagy flux under starvation. The non-acetylated form, GFP-Atg8^K13R^, showed accelerated in the protein movement into vacuoles and degradation, in comparison with wild-type Atg8 (Fig. 5f–h). A higher number of green fluorescent puncta were observed in the GFP-Atg8^K13R^ mutant than in GFP-Atg8 in the presence of bafilomycin A1 and either rapamycin treatment or non-treated controls (Figure S5d-e). The results indicated that the autophagy flux was stimulated. Furthermore, we analyzed the formation of autophagic structures in the above strains and the control Δ*atg8* mutant by transmission electron microscopy (TEM). Autophagic structures were clearly accumulated in the wild-type strain after starvation for 4 h (MM-N), but were rarely observed when cultured in the nutrient-rich medium (CM). Nevertheless, more autophagic structures were observed in the case of Atg8^K13R^, even in CM (Fig. 5i). There was no visible autophagic structure in the Δ*atg8* mutant under both conditions (Figure S5f). Thus, we conclude that Gcn5 acetylates Atg8 at K13, which leads to retention of Atg8 in the nucleus, and subsequently suppression of the autophagy flux. We further predict that this process is reversed during autophagy induction due to Gcn5 degradation upon TOR inactivation.

### Atg8 is partially deacetylated by lysine deacetylase Hdf1

Since lysine acetylation is a reversible post-translational modification, we reasoned that specific lysine deacetylases (KDACs) may be responsible for Atg8 deacetylation. A total of 10 putative genes encoding KDAC were identified in the *F. graminearum* genome (Table S4). To determine which KDAC could be involved in Atg8 deacetylation, we constructed deletion mutants for each of these genes in the GFP-Atg8-expressing strain and detected free GFP from cleavage of GFP-Atg8 after starvation. In all tested mutants, Δ*hdf1* showed a lower ratio of free GFP than the wild type, indicating that Hdf1 may be a potential KDAC for Atg8 deacetylation to regulate autophagy flux (Figure S6a).

**Fig. 6:**
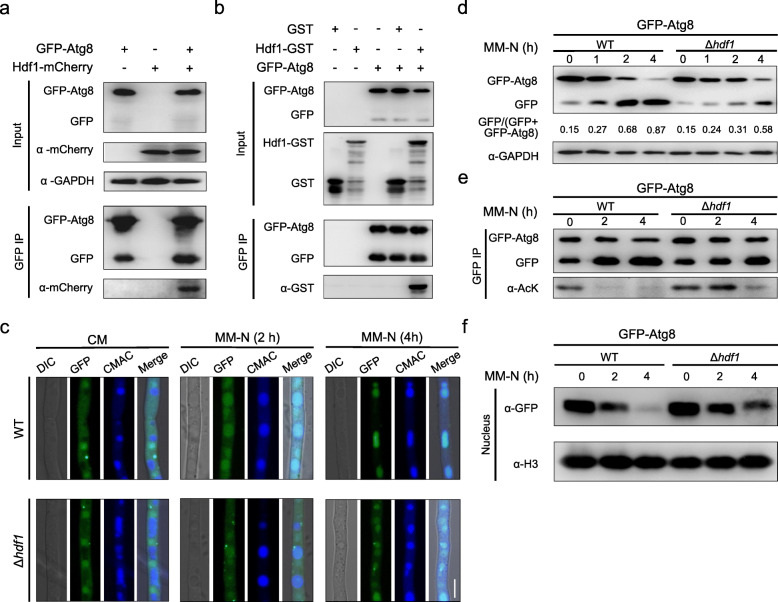
Atg8 is partially deacetylated by the deacetylase Hdf1. **a** Co-immunoprecipitation of GFP-Atg8 and mCherry-Hdf1. **b** In vitro pull-down of GST-Hdf1 and GFP-Atg8. Purified GST-Hdf1 was incubated with the lysate of the fungal strain expressing GFP-Atg8. GFP-Atg8 was immunoprecipitated with an anti-GFP agarose, the precipitated proteins were detected with anti-GFP and anti-GST antibodies. **c**, **d** Autophagy process in WT and Δ*hdf1* mutant. **c** Translocation of GFP-Atg8 in mycelia of PH-1 and Δ*hdf1* under starvation. Vacuoles were stained with the CMAC dye. Bar = 10 μm. **d** The cleavage of GFP-Atg8 in PH-1 and Δ*hdf1* under starvation. **e** Acetylation of GFP-Atg8 in PH-1 or Δ*hdf1.* GFP-Atg8 was immunoprecipitated from mycelial lysate of PH-1 or Δ*hdf1* under CM or MM-N. Acetylation level of GFP-Atg8 was detected with the anti-acetyl-lysine (α-AcK) antibody. **f** The amount of Atg8 protein in nuclear fraction of PH-1 and Δ*hdf1* under CM or MM-N. Nuclear fraction was prepared and subjected to immunoblot assay with the anti-GFP antibody. Hybridization with anti-H3 antibody served as an internal control of nuclear protein sample

To examine possible Hdf1–Atg8 interactions, mCherry-Hdf1 was co-expressed with GFP-Atg8 in the wild-type strain. Co-IP using an anti-GFP antibody followed by immunoblotting with an anti-mCherry antibody revealed that Hdf1 indeed interacts with Atg8 (Fig. 6a). In addition, recombinant glutathione S-transferase (GST)-Hdf1 purified from *E. coli* was also found to co-precipitate with GFP-Atg8 isolated from hyphae (Fig. 6b), further supporting a direct interaction between Hdf1 and Atg8. Next, we explored the role of Hdf1 in autophagy flux under starvation or rapamycin treatment. We found that disruption of Hdf1 substantially slowed down the autophagy process (Fig. 6c, d, S6b-c). To further examine the activity of Hdf1 on Atg8 deacetylation, we compared the acetylation level of Atg8 in the wild type and Δ*hdf1* during autophagy. As shown in Fig. 6e, the acetylation level of Atg8 was increased in Δ*hdf1* at various time points under starvation compared to the wild type. Given that the acetylation of Atg8 helps retain its localization in the nucleus and suppresses autophagy (Fig. 4), increased acetylation of Atg8 in Δ*hdf1* may increase Atg8 accumulation in the nucleus. Therefore, we compared the relative amount of nucleus-localized Atg8 in Δ*hdf1* and the wild type under starvation. As expected, disruption of *HDF1* resulted in significantly more accumulation of Atg8 in the nucleus than that in the wild type (Fig. 6f), where the transcription of *HDF1* demonstrated no significant change in the wild type under the same conditions (Figure S6d). Taken together, our data indicated that Hdf1 is one of the KDACs responsible for Atg8 deacetylation.

### Autophagy homeostasis is involved in fungal survival during BFIs and virulence

**Fig. 7:**
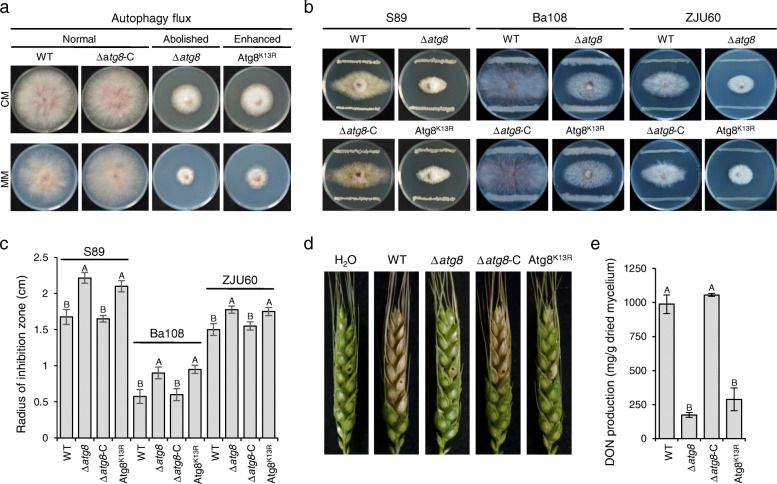
Autophagic homeostasis is critical for fungal growth, microbial competition, and virulence in *F. graminearum*. **a** Colony morphology of PH-1, Δ*atg8*, and Δ*atg8-C* and substitution mutant Atg8^K13R^ on CM, minimal medium (MM), and CM supplemented with rapamycin. The inoculated plates were imaged after 3 days of incubation. **b** Fungal sensitivity towards biocontrol bacterial strains *S. hygroscopicus* S89, *Bacillus altitudinis* Ba108, and *Pseudomonas chlororaphis* ZJU60. The dual culture plates were incubated at 25 °C for 6 days until the radius of inhibition zone did not change. **c** Radius of inhibition zone in (**b**). **d** Virulence of indicated strains on wheat heads. Infected wheat heads were examined at the 15th day post inoculation. The inoculated site on each wheat head was labeled with a black dot. **e** The deoxynivalenol (DON) production of WT, Δ*atg8* and Atg8^K13R^ at the 7th day post incubation in the toxin-inducing (TBI) medium. Line bars indicate standard deviations of three repeated experiments. The same letter on the bars indicates no significant difference according to the LSD test at *P* = 0.01

We further investigated the biological function of Atg8 acetylation by analyzing and comparing vegetative growth, pathogenesis, and stress response in WT, Δ*atg8*, Atg8^K13R^ (Δ*atg8* complemented with *Atg8*^K13R^), and Δ*atg8*-C (Δ*atg8* complemented with wild type *Atg8*). The Δ*atg8* mutant exhibited significantly slower radial growth and a reduction in the formation of aerial hyphae on the tested agar media, compared with the wild type (Fig. 7a). The reduction of radial growth and aerial hyphae were fully restored in Δ*atg8*-C, confirming that growth defects were indeed caused by the loss of Atg8 function. Interestingly, Atg8^K13R^ showed similar radial growth defects and colony morphology to that of Δ*atg8* (Fig. 7a), suggesting an essential role of K13 acetylation in Atg8 in growth. Furthermore, both Δ*atg8* and Atg8^K13R^ displayed increased fungal sensitivity towards biocontrol bacterial strains *S. hygroscopicus* S89, *Bacillus altitudinis* Ba108, and *Pseudomonas chlororaphis* ZJU60 during bacterial-fungal interactions (BFIs) (Fig. 7b, c). This indicates that autophagy homeostasis plays an important role in fungal survival during BFIs.

In addition, infection assays with wheat heads showed that virulence of both Δ*atg8* and Atg8^K13R^ was significantly reduced. On wheat heads inoculated with the wild-type and Δ*atg8*-C, scab symptoms first developed on the inoculated spikelets and rapidly spread to the whole wheat head after 15 days of inoculation. In contrast, the hyphal growth of Δ*atg8* and Atg8^K13R^ failed to spread from the inoculated floret to neighboring spikelets and subsequently caused scab symptoms only in the inoculated spikelet (Fig. 7d). DON was described as a critical virulence factor for fungal spread in wheat spikes [48]; thus, we wondered whether DON biosynthesis might be impaired in Δ*atg8* and Atg8^K13R^. To test this, we quantified the DON production of all strains in the DON-inducing medium. As expected, DON production by both Δ*atg8* and Atg8^K13R^ was significantly reduced when compared to the wild type and Δ*atg8*-C (Fig. 7e). The wild type, Δ*atg8*-C, and Atg8^K13R^ showed normal, missing, and enhanced autophagy flux, respectively. The overall results suggest that Atg8 acetylation-mediated autophagy homeostasis is critical for fungal growth, survival in BFIs as well as virulence in *F. graminearum*.

## Discussion

In this study, we present a yet unreported post-translational regulation in bacteria–fungi interactions that is part of intra-microbiome regulation of phytopathogens. The bacterial antagonist *S. rapamycinicus* S89 was shown to secrete rapamycin to inactive the target of rapamycin (TOR) pathway, which subsequently induces fungal autophagy through Atg8 acetylation caused by Gcn5 turnover in *F. graminearum*. Rapamycin was shown to be a powerful modulator of bacteria-fungi interactions and to potentially maintain microbial homeostasis in healthy wheat plants. It should be noted that the implemented wheat plants were insensitive to rapamycin (Figure S7), indicating that *S. rapamycinicus* S89 is a potential biocontrol agent for controlling Fusarium head blight. Although TOR is conserved in eukaryotes, the TOR complex subunits and downstream signaling pathways may differ in different organisms [49, 50]. This might be connected to differences in sensitivity between fungi and plants. Our finding is in line with the less known role of antibiotics in the environment; they are important signaling molecules and therefore widespread in natural habitats with high microbial diversity [51]. Former studies showed mainly direct antibiosis for *Streptomyces*–fungi interactions [52]. So far, only a few studies have reported the involvement of epigenetic regulation and post-translational regulation in bacterial–fungal interactions (BFIs) [13, 14, 53]. A forgoing study showed that *Streptomyces rapamycinicus* alters the secondary metabolism of *Aspergillus nidulans* via fungal chromatin modifying complex Saga/Ada-mediated histone acetylation [13, 53]. Another study reported that phenazine-1-carboxamide secreted by the biocontrol agent *Pseudomonas chlororaphis* ZJU60 directly affects the activity of histone acetyltransferase (HAT) Gcn5 and regulates the growth and virulence of *F. graminearum* [14]. Cumulatively, the present results and those of the forgoing studies provide important insights into the complex mechanisms of intra-microbiome regulation of fungal phytopathogens.

Increasing evidence indicates that TOR regulates various biological processes by mediating acetylation of histone and non-histone proteins in various eukaryotic cells [54–56]. In addition to forgoing observations, we observed that inactivation of TOR by rapamycin or nitrogen starvation substantially reduced the global acetylome in *F. graminearum*. However, different from previous findings, the obtained genetic and biochemical evidence strongly suggests a novel regulation that TOR inhibition promotes the degradation of HAT Gcn5 through 26S proteasome, which consequently leads to a generally decreased acetylome. To our knowledge, this is the first study suggesting that the TOR signaling pathway regulates acetylation by stimulating the degradation of a key acetyltransferase. The underlying mechanism of how inhibition of TOR accelerates Gcn5 degradation through the 26S proteasome is still unclear and needs further investigation.

Atg8/LC3 is a key autophagy-related protein involved in both autophagosome formation and autophagy cargo recruitment [57]. Its activity is regulated by various post-translational modifications, including lipidation, phosphorylation, and acetylation [57, 58]. In mammals, LC3 is acetylated by HAT p300 and deacetylated by HDAC Sirt1; the acetylation regulates LC3 translocation between the nucleus and cytoplasm and subsequently affects autophagic processes [52]. In this study, we revealed that in the plant pathogenic fungus *F. graminearum*, site-specific acetylation of Atg8 by Gcn5 also plays an important role in autophagy. In a nutrient-rich medium, Atg8 is acetylated at its K13 and the acetylated protein is retained in the nucleus to repress autophagy. The deletion mutation Δ*gcn5* substantially promotes the autophagy flux, while overexpression of *GCN5* slows down the autophagy flux. In addition, inhibition of Gcn5 degradation by MG132 treatment or deleting proteasome subunit gene *26S-R10* substantially suppressed the autophagy flux induced by rapamycin (Figure S8). We also found that the HDAC Hdf1 is partially responsible for deacetylating Atg8. Our results suggest that the acetylation status of Atg8/LC3 regulating the autophagic process might be conserved in fungi and mammals. However, the key acetylation sites in Atg8/LC3 differ among organisms.

**Fig. 8:**
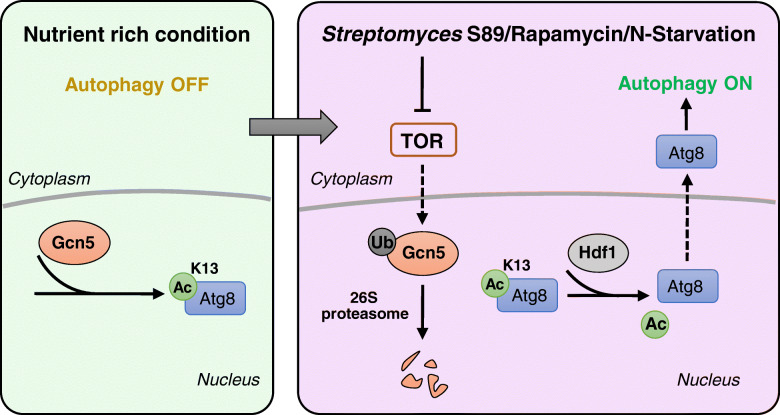
Schematic model for autophagy regulation in the *Streptomyces* S89–*F. graminearum* inter-kingdom interaction. Acetyltransferase Gcn5 acetylates Atg8 at K13 and retains Atg8 in the nucleus to prevent autophagy under nutrient-rich conditions. Upon co-cultivation with *Streptomyces* S89 and its secreted rapamycin, or nitrogen starvation, TOR inactivation promotes the degradation of Gcn5 through 26S proteasome, which reduces acetylation level of Atg8. Deacetylated Atg8 is translocated into the cytoplasm and subsequently triggers the autophagic process. Moreover, the lysine deacetylase Hdf1 deacetylates Atg8 and enhances autophagy. Autophagic homeostasis is critical for fungal growth, stress response, and virulence for *F. graminearum*

LC3 is acetylated at its K49 and K51 residues by p300 in mammal cells [26]. Although these two sites are conserved in the fungal Atg8, our results indicate that these two lysine sites are not acetylated in the Atg8 proteins purified from the mycelia of *F. graminearum*. Instead, Atg8 is mainly acetylated at K13 by Gcn5. Similar to the biological function of K49 and K51 acetylation in LC3, K13 acetylation in Atg8 results in its retention in the nucleus, while the substitution mutant Atg8^K13R^ is exported from the nucleus and accelerates the autophagy flux. K13 is evolutionarily conserved in Atg8 proteins in fungi and insects, but not mammals. The mechanism of autophagy regulation by Gcn5-mediated Atg8 acetylation at K13 (Fig. 8) might also be analogous in other fungi, as well as insects, since deletion of *GCN5* in the filamentous fungus *Magnaporthe oryzae* and *Drosophila* also promoted autophagic flux [59, 60]. Findings in this study therefore extend our understanding of the interplay between epigenetic regulation and autophagy.

Autophagy is involved in various developmental processes and human diseases [61]. Regulating autophagic activity has thus been considered a potential therapeutic target for autophagy-related diseases [62]. Several drugs and small molecules targeting the autophagy flux have been developed, such as bafilomycin A1, chloroquine, and hydroxychloroquine [63, 64]. Autophagy is also important for pathogenesis, virulence, and stress response in fungi. Abolishment of autophagy by deleting *ATG*s in plant pathogenic fungi substantially reduced fungal sexual and asexual development and virulence [45, 59, 65, 66]. In the present study, we found that not only autophagy defective mutants but also transformants with enhanced autophagy were attenuated in virulence of *F. graminearum*. Moreover, these mutants were more sensitive towards the biocontrol agents *S. hygroscopicus* S89, *B. altitudinis* Ba108, and *P. chlororaphis* ZJU60.

Altogether, our data suggest that autophagy homeostasis regulated by Atg8 acetylation is essential for *F. graminearum* growth, virulence, and survival in the plant microbiota. Moreover, we show that the autophagic process is a potential biocontrol target for Fusarium head blight. Harnessing the plant-associated microbes or microbe-secreted active compounds to target the fungal autophagy flux will provide new opportunities for the prevention and control of fungal diseases.

## Conclusions

Autophagy homeostasis plays an essential role in fungal growth and competition, as well as for virulence. Our work reveals a novel post-translational regulation of autophagy initiated by a bacterial antibiotic. Rapamycin was shown to be a powerful modulator of bacteria–fungi interactions with potential importance in explaining microbial homeostasis in healthy plant microbiomes. The autophagic process provides novel possibilities and targets to biologically control pathogens.

## Supplementary Information

**Additional file 1: Table S1.** Antagonistic activity of *Streptomyces* strains isolated in this study. **Table S2.** *ATG* gene expression identified by RNA-Seq analysis under nitrogen starvation in *Fusarium graminearum.* **Table S3.** List of identified Gcn5-interacting proteins associated with autophagy. **Table S4.** Putative histone deacetyltransferases (HDACs) in *F. graminearum.* **Table S5.** PCR primers used in this study.

**Additional file 2: Figure S1.** Rapamycin or nitrogen starvation effectively induce autophagy and reduce global acetylome in *F. graminearum*. (a) GFP-Atg8 localization patterns in the wild type strain PH-1 grown in completed medium (CM). Bar = 10 μm. Vacuoles were stained with the CMAC dye. (b) Mass spectrometry data of rapamycin purified from S89 supernatant. The rapamycin producing model strain *S. hygroscopicus* NRRL5491 and pure rapamycin were used as positive control samples. (c) Autophagy flux of the fungus upon rapamycin or MM-N treatment. GFP-Atg8 labeled PH-1 was grown in CM before rapamycin or MM-N treatment. (d) Representative lysine acetylome profiling of *F. graminearum* upon nitrogen starvation. Total proteins were extracted from fresh mycelia and analyzed by immunoblot using an anti-acetyl-lysine antibody. Hybridization with an anti-GAPDH antibody served as an internal control. The asterisk indicates acetylated histones. **Figure S2.** 3-MA treatment inhibits autophagy in *F. graminearum*. (a) GFP-Atg8 labeled PH-1 was grown in complete medium (CM) and treated with rapamycin in the presence/absence of 3-MA. Autophagic bodies in vacuoles were stabilized by the inhibitor bafilomycin A1. Vacuoles were stained with the CMAC dye. Bar=10 μm. (b) Quantification of the GFP-Atg8 puncta in (a). The data are presented as mean ± s. d., *n* = 30 compartments. **, *P* < 0.01. (c) Autophagy flux of the WT strain in the presence/absence of 3-MA upon rapamycin treatment. **Figure S3.** Deletion and *in locus* overexpression of *GCN5*, and autophagy flux of the wild-type, Δ*gcn5* or OE-*GCN5* upon rapamycin treatment. (a) Diagram of *GCN5* deletion and *in locus* overexpression. F1 marked the forward primer for identification of genetic transformants of Gcn5. R1 and R2 are the designed reverse primers for identification. (b) Identification of *Δgcn5* and OE-*GCN5* strains by PCR assay. M: marker. (c) Relative mRNA level of *GCN5* in the wild-type PH-1 (WT) or OE-*GCN5* strain. Total RNA extracted from PH-1 or OE-*GCN5* was subjected to a qRT-PCR assay. The expression of *ACTIN* in each sample was used as a reference. (d) Autophagy flux of the WT, Δ*gcn5* and OE-*GCN5* upon rapamycin treatment. **Figure S4.** The acetyltransferase activity of Gcn5 is critical for regulating autophagy. (a) Alignment of protein sequences of Gcn5 orthologs from various fungal species. The position highlighted in red indicates conserved 130th glutamic acid in *F. graminearum*. (b) GFP-Atg8 punctum formation in different strains during autophagy induced by rapamycin. Vacuoles were stained with the CMAC dye. Bar=10 μm. (c) Quantification of GFP-Atg8 punctum occurrence per compartment in (b). The data are presented as mean ± s. d., *n* = 30 compartments. **, *P* < 0.01. **Figure S5.** K13 but not K38 acetylation in Atg8 is involved in autophagy. (a) Alignment of the protein sequences of Atg8 orthologs from various species. The positions highlighted in red indicate acetylated K13 and K38 lysine residues identified in Atg8 in *F. graminearum*. (b) Cellular localization of GFP-Atg8^K38R^. Mycelia of the GFP-Atg8^K38R^-expressing strain were grown in CM. H1-mCherry was used as a nuclear marker. Bar=10 μm. (c) Immunoblot assay of the cleavage of GFP-Atg8^K38R^ or GFP-Atg8 upon nitrogen starvation. (d) GFP-Atg8 punctum formation in different strains during autophagy induced by rapamycin. Vacuoles were stained with the CMAC dye. Bar=10 μm. (e) Quantification of GFP-Atg8 punctum occurrence per compartment in (d). The data are presented as mean ± s. d., *n* = 30 compartments. **, *P* < 0.01. (f) TEM images of the autophagic structures in Δ*atg8* mutant under nitrogen starvation. **Figure S6.** Deacetylase Hdf1 plays a positive role in autophagy regulation. (a) Autophagy flux of various deletion mutants of deacetylases in *F. graminearum*. Total proteins extracted from the mycelia of various mutants grown in CM (indicated as 0 h) or MM-N for 4 h were analyzed by immunoblot assays with the anti-GFP antibody. The extent of autophagy was estimated and indicated underneath the blot. The intensities of bands were quantified with ImageJ. Hybridization with the α-GAPDH antibody served as an internal control. (b) GFP-Atg8 punctum formation in wild type PH-1 and Δ*hdf1* during autophagy induced by rapamycin. Vacuoles were stained with the CMAC dye. Bar=10 μm. (c) Quantification of GFP-Atg8 punctum occurrence per compartment in (b). The data are presented as mean ± s. d., *n* = 30 compartments. **, *P* < 0.01. (d) Relative mRNA level of *HDF1* in CM or MM-N at the indicated time points. Total RNA was extracted from the mycelia and subjected to qRT-PCR assay. The expression of *ACTIN* in each sample was used as a reference. **Figure S7.** Wheat plants are insensitive to rapamycin. (a) Representative wheat seedlings on the 4^th^ day post-rapamycin treatment. Germinated wheat seeds were grown in a light-dark (12/12) growth chamber after rapamycin (25 μM) treatment. Diluted DMSO was used as non-treatment control. Bar=1 cm. (b) Quantification of the length of seedlings in (a). The data are presented as mean ± s. d., *n* =50. **Figure S8.** Inhibition of ubiquitin-proteasome system suppresses autophagy flux induced by rapamycin in *F. graminearum*. (a) GFP-Atg8 translocation during autophagy induced by rapamycin in the wild type PH-1 in the presence/absence proteasome inhibitor MG132, and 26S proteasome defective mutant Δ*26S-RS10* in *F. graminearum*. Vacuoles were stained with the CMAC dye. Bar=10 μm. (b) Autophagy flux detected with a GFP cleavage assay in different strains or treatments. (c) GFP-Atg8 punctum formation in wild type PH-1, Δ*26S-RS10* or MG132 treated PH-1 during autophagy induced by rapamycin. Bar=10 μm. (d) Quantification of GFP-Atg8 punctum occurrence per compartment in (c). The data are presented as mean ± s. d., *n* = 30 compartments. **, *P* < 0.01. **Figure S9.** Linear range of western blot bands. Total protein lysates of the wild type strain labeled with GFP-Atg8 were quantified with a BCA protein assay kit, and then subjected to western blotting in a volume range from 1 to 12 μL. The protein GAPDH (a, b), GFP-Atg8 (c, d), and H3 (e, f) were detected with corresponding antibodies, the intensities of specific bands were quantified with ImageJ and analyzed with linear regression.

## Data Availability

No obtained data required submission to a public repository. Raw data or further details related to the conducted experiments can be obtained upon request from the corresponding author.
