# Supplementary material for: Post-translational regulation of autophagy is involved in intra-microbiome suppression of fungal pathogens
Source: Microbiome. 2021 Jun 6;9:131. doi: 10.1186/s40168-021-01077-y (PMC8182927; doi:10.1186/s40168-021-01077-y)
Supplement: Supplementary file 2 — Additional file 1: Table S1. Antagonistic activity of Streptomyces strains isolated in this study. Table S2. ATG gene expression identified by RNA-Seq analysis under nitrogen starvation in Fusarium graminearum. Table S3. List of identified Gcn5-interacting proteins associated with autophagy. Table S4. Putative histone deacetyltransferases (HDACs) in F. graminearum. Table S5. PCR primers used in this study. [file 40168_2021_1077_MOESM2_ESM.docx]

**Post-translational regulation of autophagy is involved in intra-microbiome suppression of fungal pathogens**

Jing Wang *et al.*

**Supplementary Information**

**Supplementary materials and methods**

**Fungal and bacterial strains**

Wild-type strain *Fusarium graminearum* PH-1 (NRRL 31084); *Streptomyces hygroscopicus* (NRRL5491); XK1-25 (Tryptophan-deficient yeast); *Escherichia coli* BL21 (CD601), *E. coli* 5α (CD201) (Beijing Transgene Biotechco., Ltd.); Y2H Gold (Clontech laboratories, Inc., USA); Other *Streptomyces* strains were isolated, identified and preserved in the frame of this study.

**Antibodies**

Mouse monoclonal-anti-GAPDH (EM1101), Mouse monoclonal-anti-GST (M0807), Goat polyclonal anti-mouse IgG-HRP (HA1006), Goat polyclonal anti-rabbit IgG-HRP (HA1001) (Hangzhou HuaAn Biotechnology co., Ltd.); Rabbit monoclonal anti-H3 (#61475), Rabbit polyclonal anti-acetyl-histone H3 (#61637), Rabbit polyclonal anti-acetyl-histone H3(lysine14) (#39599), Rabbit polyclonal anti-acetyl-histone H3(lysine27) (#39135) (Active Motif, La Hulpe, Belgium); Rabbit polyclonal anti-GFP (ab32146), Mouse monoclonal anti-6×His (ab18184), Rabbit monoclonal anti-acetyl-histone H2B(lysine11) (ab40975) (Abcam, Cambridge, USA); Mouse monoclonal anti-Flag (SL5142) (Sigma, Burlington, USA); Mouse monoclonal anti-acetylated-lysine (sc-81623) (Santa Cruz Biotech, Heidelberg, Germany); Anti-GFP agarose (GTA10) (ChromoTek, Munich, Germany), Ni sepharose 6 fast flow (11-0008-87)(GE healthcare, Chicago, USA), GST Agarose (#20211) (Thermo Fisher Scientific, Waltham, USA).

**Chemicals and reagents**

Rapamycin (HY10219), MG132 (HY13259), 3-MA (3-Methyladenine, HY19312), Bafilomycin A1 (HY100558), (MedChem Express co., Ltd); PMSF (Phenylmmethanesulfonyl fluoride, #36978) (Thermo Fisher Scientific, Waltham, USA); CHX (Cycloheximide, C104450), Driselase (D9515) (Sigma,Burlington, USA), IPTG (Isopropyl β-D-Thiogalactoside, A100487), Acryl/Bis 40% solution (37.5:1) (B546014), 4×Tris-HCl/SDS pH 6.8/8.8 (B546022, B542021), TEMED (N,N,N’,N’-Tetramethylethylenediamine, A100761), Imidazole (A500529), Fungi and yeast protease inhibitor complex (C500027) (Sangon Biotech (Shanghai) co., Ltd.); APS (Ammonium persulphate, FD2050) (Fude Biotechnology co., Ltd.); PCN (phenazine-1-carboxamide, SF160309232) (Qinba chemical co., Ltd.); Lysozyme (RM1027) , Cellulose (RM1030) (Hangzhou RYON biotech, co., Ltd).

**Culture medium**

ISP2 agar (1% Malt Extract, 0.4% Yeast Extract, 0.6% D-Glucose, 2% Agar, pH 7.2); CM agar (0.1% Casamino acid, 1% D-Glucose, 0.2 % Peptone, 0.1% Yeast Extract, 0.6% NaNO_3_, 0.052% KCl, 0.052% MgSO_4_·7H_2_O, 0.152% KH_2_PO4, 0.1% v/v Vitamin solution (0.01% Biotin, 0.01% Pyridoxin, 0.01% Thiamine, 0.01% Riboflavin, 0.01% p-aminobenzonic acid, 0.01% Nicotinic acid) and 0.1% v/v Trace elements (2.2% ZnSO_4_·7H_2_O, 1.1% H_3_BO_3_, 0.5% MnCl_2_·4H_2_O, 0.5% FeSO_4_·7H_2_O, 0.17% CoCl_2_·6H_2_O, 0.16% CuSO_4_·5H_2_O, 0.15% Na_2_MoSO_4_·5H_2_O, 5% Na_4_EDTA).

**Strain construction**

The gene deletion and complementation mutants of *F. graminearum* were constructed using a polyethylene glycol (PEG)-mediated protoplast transformation method. To prepare protoplasts of *F. graminearum*, fresh mycelia of each strain were treated with 0.7 M sodium chloride solution dissolved driselase, lysozyme, and cellulose. Primers used to amplify the flanking sequences for each gene are listed in Table S5. For gene complementation, taking Atg8 as an example, a GFP-Atg8 fusion fragment was obtained using the double-joint PCR approach, and then co-transformed with *Xho*I-digested *p*YF11- gfp-g418 plasmid into the yeast strain XK1-25 using the Alkali-Cation Yeast Transformation Kit (MP Biomedicals, Solon, USA) to generate a GFP-Atg8 fusion vector. Gcn5-GFP, Gcn5-mCherry, H1-mCherry, and Hdf1-mCherry vectors were constructed with a similar strategy. Recombined vectors were transferred into the protoplasts of each corresponding deletion mutant. Transformants were identified by PCR and western blot assays. The *gpda*-mediated over-expression of *GCN5* was performed as described below. Upstream and downstream fragment of the promoter of *GCN5*, the open reading frame (ORF) of *GCN5* were amplified from DNA of *F. graminearum*, GFP and G418 coding sequence were amplified from the *p*YF11-gfp-g418 vector, and the *Aspergillus nidulans* promoter *gpda* (glyceraldehyde 3-phosphate) was amplified from the DNA of *A. nidulans*. The six fragments were constructed with a multi-joint PCR. Then the long-fragment was transferred into the protoplasts of Δ*gcn5*. Transformants were verified by PCR for the effective *in locus* promoter replacement and RT-qPCR for the over-expression efficiency.

For *in locus* site mutation, a gene replacement cassette carrying the selective markers hygromycin (HPH), geneticin (G418), or nourseothricin (NTC), the native promoter and flanking sequences containing the to-be-replaced amino acid sites of each gene and the coding sequence of fluorescent protein genes were amplified and constructed by multi-joint PCR. The joint long fragments were transformed into the protoplasts of corresponding gene deletion mutants. The fragments containing to-be-mutated sites were sequenced after PCR amplification. All primers used in this study were listed in Table S5.

**Recombinant protein purification**

Recombinant Atg8-6×His, Gcn5-6×His and GST-Hdf1 were expressed in *E. coli* BL21. Expressions of recombinant proteins were induced with 1mM IPTG at 25 °C for 4 h, then cells were collected and re-suspended with 1×TBS containing 1mM PMSF and 5% glycerol, followed by ultrasonication. His-Tag labeled proteins were purified with Ni sepharose 6 fast flow, eluted with gradient concentrations of imidazole and dialyzed with Slide-A lyzer Dialysis Cassettes against 20 mM Tris-HCl at pH 8.0, 150 mM NaCl and 5% glycerol. GST-Hdf1 was purified using glutathione-sepharose 4B beads, followed by five times washing with 1×TBS buffer. Then, proteins were eluted with 10 mM Glutathione dissolved in 1×TBS and dialyzed.

**Pull-down assay**

Recombinant 6×His-Atg8 or GST-Hdf1 proteins that were purified from bacteria were incubated with total mycelial lysate of Gcn5-GFP or GFP-Atg8 expressing *F. graminearum* at 4 °C for 4 h, then anti-GFP agarose was added and another 2-h incubation was conducted. The immunocomplexes were washed six times with washing buffer (50 mM Tris-HCl pH 8.0, 150 mM NaCl, 1% NP40) and subjected to immunoblot analysis.

**Cytoplasmic/nuclear fractionation assays**

Freshly prepared mycelia (0.2 g) of each strain were harvested and ground to a fine powder in liquid nitrogen. They were mixed with 5 mL/g of lysis buffer (20 mM Tris-HCl, pH 7.5, 20 mM KCl, 2 mM EDTA, 2.5 mM MgCl_2_, 25% glycerol, 250 mM Sucrose, and 5 mM DTT) supplemented with a protease inhibitor cocktail. The homogenate was filtered through a double layer of Miracloth. The flow-through was centrifugated at 1 500 *g* for 10 min, and the supernatant, consisting of the cytoplasmic fraction, was centrifuged at 10 000 *g* for 10 min at 4°C and subsequently collected. The pellet was washed six times with 1 mL of nuclear resuspension buffer NRB1 (20 mM Tris-HCl, pH 7.4, 25% glycerol, 2.5 mM MgCl_2_, and 0.2% Triton X-100) and then resuspended with 500 μL of NRB2 (20 mM Tris-HCl, pH 7.5, 0.25 M Sucrose, 10 mM MgCl_2_, 0.5% Triton X-100, and 5 mM β-mercaptoethanol) supplemented with a protease inhibitor cocktail and carefully overlaid on top of 500 μL NRB3 (20 mM Tris-HCl, pH 7.5, 1.7 M Sucrose, 10 mM MgCl_2_, 0.5% Triton X-100, and 5 mM β-mercaptoethanol) supplemented with the protease inhibitor cocktail. These were centrifuged at 16 000 *g* for 45 min at 4 °C. The final nuclear pellet was resuspended in 400 μL lysis buffer. For quality controls of the fractionation, GAPDH protein was detected and used as a cytoplasmic control and histone H3 was used as a nuclear control and marker. To verify that the detected bands are within the linear range, protein concentrations were determined with the BCA protein assay kit (A53226; Thermo Fisher Scientific, USA). Then, a volume range of 1-12 μL mycelial lysate was loaded, used for quantification of the detected bands and subsequent linear regression analysis as shown in Fig. S9.

***In vitro* acetylation assay**

For the *in vitro* acetylation assays, purified Atg8-6×His (10 μg) was incubated with recombinant Gcn5-6×His or Gcn5-GFP/Gcn5-3×Flag immunoprecipitated from fungal lysate in the presence of acetyl-coenzyme A (100 μM) and 10 μL 5×HAT buffer (250 mM Tris-HCl, pH 8.0, 250 mM KCl, 0.5 mM EDTA, 5 mM dithiothreitol, 5 mM protease inhibitor, 25% glycerol, 5 mM sodium butyrate) in a total volume of 50 μL. The suspensions were gently mixed every five minutes and incubated at 37 °C for 45 min. The reactions were stopped by the addition of 12.5 μL 5×protein loading buffer and then boiled for 5 min. Reaction products were separated by SDS-PAGE and immunoblotted with the aforementioned antibodies, the acetylated Atg8 or Atg8^K13R^ proteins were determined with the anti-acetyl-lysines antibody.

**Mass spectrometry**

To identify interacting proteins of Gcn5, mycelia of *in locus* *gpda*-mediated over-expressed GFP-tagged Gcn5 were cultivated in liquid CM, then mycelia were collected and lysed with extraction buffer described in the immunoprecipitation module. The extracts were immunoprecipitated with anti-GFP agarose. The washed agarose was resuspended with elution buffer (1% SDS, 20 mM Tris-HCl at pH 7.4, 150 mM NaCl) and boiled for 10 min. Then the precipitate was centrifuged at 12 000 *g* for 10 min at room temperature. The supernatant was carefully removed. Subsequently, 3 μL was subjected to an immunoblot to test the immunoprecipitation efficiency. The rest was subjected to MS analysis (Applied Protein Technology, Shanghai, China). To identify the acetylated sites of Atg8 in wild type and Δ*gcn5* strains, GFP-tagged Atg8 proteins of these two strains were cultivated and immunoprecipitated as Gcn5-GFP. The eluted material was denatured and resolved by 12% SDS-PAGE. The Coomassie blue stained GFP-Atg8 bands were excised from the gel and subjected to tryptic digestion and mass spectrometry (Beijing Biotech-Pack Scientific). Protein and modifications were explored with database searches and peptide identifications were validated with Peptide Prophet.

**RNA isolation and qPCR analyses**

Total RNA was isolated using RNAiso Plus (#9018) (TaKaRa Bio Inc. Japan). One microgram of the total RNA was reversely transcribed into cDNA using the HiScriptII 1st Strand cDNA Synthesis Kit (Vazyme Biotech Co., Ltd.). Quantitative real-time PCR analysis was performed using HiScriptII Q select RT SuperMix for qPCR (Vazyme Biotech Co., Ltd.) and the real-time PCR instrument CFX-96 (Bio-Rad). The relative mRNA levels of target genes were calculated from the corresponding standard curve with the instruemnt software and normalized based on *ACTIN* expression in the same samples.

**CMAC and DAPI staining**

Freshly prepared mycelia of each sample were incubated in 0.05 mM CMAC at room temperature and protected from light for ~30 min for vacuole tracking; for nuclear staining, each sample was soaked in 0.1 μg/L DAPI solution for ~30 min at room temperature, after that, samples were washed and observed under a confocal microscope (Zeiss LSM 780).

**Pathogenicity and DON** **biosynthesis assays**

Pathogenicity and DON biosynthesis assays were performed as before (1). An aliquot of 10 μL conidial suspension at a concentration of 1×10^5^ conidia mL^-1^ from each strain was injected into the middle spikelet of flowering wheat heads. Twenty individual wheat heads were inoculated for each tested strain. The pathogenicity assay was kept at a humidity of 95-100%. Images were taken 15 days after inoculation and the disease index was calculated. To quantify DON production in the wild type, mutants were cultured in TBI (Trichothecenes Biosynthesis Inducing) liquid medium, DON was extracted from each strain after incubation at 28 °C for 7 days. The cell-free supernatant was filtered and passed through a SampliQ Amino (NH2) solid phase extraction column (Agilent Technologies). Subsequently, 4 mL of the purified extract were dried under a nitrogen stream. The residue was dissolved in 1 mL methanol: water (40:60, v/v), followed by centrifugation at 10, 000 *g* and subsequently analyzed by LC-MS/MS.

**Plant growth conditions**

To determine the effect of rapamycin on the growth of wheat plants, sterilized wheat seeds (Cultivar: Jimai 22) were subjected to cold treatment in sterilized water supplemented with rapamycin (25 μM) at 4°C in the dark for 3 days. After germination, the germinated seeds were randomly selected and placed on sterilized filter paper in a petri dish. Then, 5 mL of rapamycin solution (25 μM) was pipetted on them. Wheat seedlings were incubated in a growth chamber at 23°C light/21°C dark, 65% humidity under a 12-h light/12-h dark photoperiod. Wheat seeds without rapamycin treatment were used as a control. After 4 days of incubation, growth of wheat seedlings was imaged, and the lengths of seedlings in each treatment were measured and statistically analyzed.

**Supplementary Tables**

**Table S1 Antagonistic activity of *Streptomyces* strains**

| Strain Number | Cluster Number | Inhibition zone repeat 1 (cm) | Inhibition zone repeat 2 (cm) | Inhibition zone repeat 3 (cm) | Inhibition zone repeat 4 (cm) | Inhibition zone repeat 5 (cm) | Average | Standard Deviation |
| --- | --- | --- | --- | --- | --- | --- | --- | --- |
| S89 | No.1 | 1.80 | 1.75 | 1.80 | 1.80 | 1.90 | 1.81 | 0.05 |
| S113 | No.1 | 1.78 | 1.72 | 1.80 | 1.73 | 1.76 | 1.76 | 0.03 |
| S95 | No.2 | 1.75 | 1.75 | 1.76 | 1.75 | 1.75 | 1.75 | 0.00 |
| S181 | No.35 | 1.80 | 1.70 | 1.80 | 1.70 | 1.70 | 1.74 | 0.05 |
| S168 | No.2 | 1.70 | 1.70 | 1.80 | 1.70 | 1.70 | 1.72 | 0.04 |
| S176 | No.12 | 1.60 | 1.60 | 1.80 | 1.70 | 1.80 | 1.70 | 0.10 |
| S88 | No.12 | 1.72 | 1.70 | 1.68 | 1.69 | 1.71 | 1.70 | 0.02 |
| S111 | No.13 | 1.70 | 1.72 | 1.75 | 1.65 | 1.68 | 1.70 | 0.04 |
| S104 | No.27 | 1.65 | 1.70 | 1.72 | 1.73 | 1.68 | 1.70 | 0.03 |
| S39 | No.7 | 1.50 | 1.60 | 1.70 | 1.70 | 1.80 | 1.66 | 0.11 |
| S85 | No.13 | 1.60 | 1.65 | 1.70 | 1.65 | 1.65 | 1.65 | 0.04 |
| S38 | No.24 | 2.00 | 1.60 | 1.70 | 1.50 | 1.40 | 1.64 | 0.23 |
| S138 | No.2 | 1.60 | 1.60 | 1.70 | 1.60 | 1.65 | 1.63 | 0.04 |
| S174 | No.2 | 1.60 | 1.60 | 1.60 | 1.65 | 1.65 | 1.62 | 0.03 |
| S171 | No.2 | 1.60 | 1.60 | 1.60 | 1.60 | 1.60 | 1.60 | 0.00 |
| S103 | No.3 | 1.60 | 1.59 | 1.60 | 1.60 | 1.60 | 1.60 | 0.00 |
| S177 | No.11 | 1.50 | 1.50 | 1.60 | 1.60 | 1.70 | 1.58 | 0.08 |
| S166 | No.2 | 1.60 | 1.55 | 1.50 | 1.60 | 1.60 | 1.57 | 0.04 |
| S108 | No.19 | 1.50 | 1.50 | 1.60 | 1.60 | 1.50 | 1.54 | 0.05 |
| S52 | No.2 | 1.50 | 1.60 | 1.50 | 1.60 | 1.50 | 1.54 | 0.05 |
| S167 | No.2 | 1.60 | 1.65 | 1.60 | 1.40 | 1.40 | 1.53 | 0.12 |
| S130 | No.2 | 1.50 | 1.50 | 1.60 | 1.50 | 1.50 | 1.52 | 0.04 |
| S182 | No.12 | 1.50 | 1.60 | 1.50 | 1.80 | 1.20 | 1.52 | 0.22 |
| S32 | No.2 | 1.50 | 1.50 | 1.55 | 1.50 | 1.50 | 1.51 | 0.02 |
| S112 | No.6 | 1.50 | 1.50 | 1.45 | 1.55 | 1.55 | 1.51 | 0.04 |
| S5 | No.16 | 1.30 | 1.70 | 1.70 | 1.40 | 1.40 | 1.50 | 0.19 |
| S15 | No.2 | 1.50 | 1.60 | 1.60 | 1.40 | 1.40 | 1.50 | 0.10 |
| S21 | No.2 | 1.60 | 1.50 | 1.50 | 1.45 | 1.45 | 1.50 | 0.06 |
| S59 | No.16 | 1.45 | 1.55 | 1.55 | 1.55 | 1.40 | 1.50 | 0.07 |
| S49 | No.2 | 1.45 | 1.45 | 1.50 | 1.55 | 1.50 | 1.49 | 0.04 |
| S11 | No.2 | 1.50 | 1.50 | 1.60 | 1.40 | 1.40 | 1.48 | 0.08 |
| S44 | No.12 | 1.30 | 1.20 | 1.50 | 1.80 | 1.60 | 1.48 | 0.24 |
| S42 | No.7 | 1.45 | 1.42 | 1.45 | 1.46 | 1.50 | 1.46 | 0.03 |
| S54 | No.2 | 1.30 | 1.40 | 1.30 | 1.60 | 1.65 | 1.45 | 0.17 |
| S48 | No.2 | 1.40 | 1.40 | 1.40 | 1.50 | 1.55 | 1.45 | 0.07 |
| S149 | No.2 | 1.40 | 1.50 | 1.50 | 1.40 | 1.40 | 1.44 | 0.05 |
| S164 | No.2 | 1.35 | 1.30 | 1.40 | 1.55 | 1.60 | 1.44 | 0.13 |
| S151 | No.2 | 1.40 | 1.50 | 1.40 | 1.40 | 1.50 | 1.44 | 0.05 |
| S170 | No.2 | 1.40 | 1.40 | 1.50 | 1.40 | 1.40 | 1.42 | 0.04 |
| S53 | No.2 | 1.30 | 1.20 | 1.30 | 1.50 | 1.50 | 1.36 | 0.13 |
| S110 | No.25 | 1.32 | 1.31 | 1.35 | 1.37 | 1.40 | 1.35 | 0.04 |
| S51 | No.2 | 1.30 | 1.40 | 1.30 | 1.40 | 1.30 | 1.34 | 0.05 |
| S139 | No.2 | 1.30 | 1.30 | 1.30 | 1.35 | 1.40 | 1.33 | 0.04 |
| S43 | No.21 | 1.32 | 1.30 | 1.31 | 1.34 | 1.35 | 1.32 | 0.02 |
| S50 | No.2 | 1.20 | 1.60 | 1.30 | 1.30 | 1.20 | 1.32 | 0.16 |
| S90 | No.26 | 1.20 | 1.30 | 1.40 | 1.40 | 1.20 | 1.30 | 0.10 |
| S141 | No.2 | 1.30 | 1.30 | 1.40 | 1.25 | 1.25 | 1.30 | 0.06 |
| S143 | No.2 | 1.20 | 1.30 | 1.30 | 1.30 | 1.25 | 1.27 | 0.04 |
| S68 | No.7 | 1.30 | 1.30 | 1.30 | 1.20 | 1.20 | 1.26 | 0.05 |
| S178 | No.7 | 1.00 | 1.20 | 1.20 | 1.50 | 1.40 | 1.26 | 0.19 |
| S13 | No.2 | 1.25 | 1.20 | 1.35 | 1.20 | 1.30 | 1.26 | 0.07 |
| S70 | No.2 | 1.23 | 1.25 | 1.26 | 1.25 | 1.30 | 1.26 | 0.03 |
| S147 | No.2 | 1.20 | 1.30 | 1.40 | 1.20 | 1.15 | 1.25 | 0.10 |
| S131 | No.2 | 1.30 | 1.25 | 1.30 | 1.20 | 1.15 | 1.24 | 0.07 |
| S23 | No.2 | 1.20 | 1.25 | 1.30 | 1.20 | 1.20 | 1.23 | 0.04 |
| S137 | No.2 | 1.25 | 1.30 | 1.30 | 1.10 | 1.20 | 1.23 | 0.08 |
| S148 | No.2 | 1.30 | 1.25 | 1.30 | 1.10 | 1.20 | 1.23 | 0.08 |
| S26 | No.2 | 1.20 | 1.15 | 1.25 | 1.20 | 1.30 | 1.22 | 0.06 |
| S78 | No.13 | 1.20 | 1.20 | 1.20 | 1.30 | 1.20 | 1.22 | 0.04 |
| S144 | No.2 | 1.15 | 1.10 | 1.30 | 1.30 | 1.25 | 1.22 | 0.09 |
| S127 | No.2 | 1.25 | 1.25 | 1.30 | 1.10 | 1.15 | 1.21 | 0.08 |
| S31 | No.2 | 1.15 | 1.20 | 1.20 | 1.25 | 1.25 | 1.21 | 0.04 |
| S175 | No.4 | 1.00 | 1.10 | 1.20 | 1.20 | 1.50 | 1.20 | 0.19 |
| S24 | No.2 | 1.10 | 1.05 | 1.10 | 1.30 | 1.40 | 1.19 | 0.15 |
| S150 | No.2 | 1.20 | 1.20 | 1.20 | 1.20 | 1.15 | 1.19 | 0.02 |
| S34 | No.2 | 1.20 | 1.10 | 1.20 | 1.20 | 1.20 | 1.18 | 0.04 |
| S20 | No.2 | 1.10 | 1.10 | 1.20 | 1.20 | 1.30 | 1.18 | 0.08 |
| S22 | No.2 | 1.20 | 1.20 | 1.20 | 1.15 | 1.15 | 1.18 | 0.03 |
| S173 | No.2 | 1.10 | 1.10 | 1.15 | 1.20 | 1.35 | 1.18 | 0.10 |
| S129 | No.2 | 1.10 | 1.15 | 1.15 | 1.20 | 1.25 | 1.17 | 0.06 |
| S132 | No.2 | 1.20 | 1.10 | 1.20 | 1.20 | 1.10 | 1.16 | 0.05 |
| S122 | No.2 | 1.10 | 1.20 | 1.30 | 1.00 | 1.20 | 1.16 | 0.11 |
| S146 | No.2 | 1.25 | 1.30 | 1.30 | 0.95 | 1.00 | 1.16 | 0.17 |
| S7 | No.2 | 1.20 | 1.20 | 1.20 | 1.05 | 1.05 | 1.14 | 0.08 |
| S41 | No.9 | 0.90 | 1.00 | 1.00 | 1.20 | 1.50 | 1.12 | 0.24 |
| S126 | No.2 | 1.05 | 1.05 | 1.10 | 1.15 | 1.20 | 1.11 | 0.07 |
| S82 | No.30 | 1.10 | 1.20 | 1.10 | 1.00 | 1.10 | 1.10 | 0.07 |
| S163 | No.2 | 1.10 | 1.00 | 1.10 | 1.10 | 1.15 | 1.09 | 0.05 |
| S64 | No.16 | 1.05 | 1.10 | 1.15 | 1.12 | 1.00 | 1.08 | 0.06 |
| S25 | No.2 | 1.00 | 1.05 | 1.10 | 1.10 | 1.15 | 1.08 | 0.06 |
| S124 | No.2 | 0.90 | 1.00 | 1.00 | 1.30 | 1.20 | 1.08 | 0.16 |
| S161 | No.2 | 1.10 | 1.10 | 1.00 | 1.00 | 1.20 | 1.08 | 0.08 |
| S1 | No.5 | 1.30 | 1.00 | 1.10 | 0.80 | 0.90 | 1.02 | 0.19 |
| S27 | No.2 | 0.95 | 0.95 | 1.00 | 1.05 | 1.10 | 1.01 | 0.07 |
| S67 | No.2 | 0.98 | 0.97 | 0.95 | 1.10 | 1.00 | 1.00 | 0.06 |
| S165 | No.2 | 1.10 | 1.10 | 0.90 | 0.90 | 1.00 | 1.00 | 0.10 |
| S16 | No.2 | 1.05 | 1.05 | 1.10 | 0.85 | 0.90 | 0.99 | 0.11 |
| S46 | No.23 | 0.80 | 1.00 | 1.20 | 0.90 | 0.90 | 0.96 | 0.15 |
| S55 | No.2 | 1.00 | 0.90 | 1.00 | 0.90 | 1.00 | 0.96 | 0.05 |
| S153 | No.2 | 1.00 | 1.10 | 1.00 | 0.70 | 0.90 | 0.94 | 0.15 |
| S172 | No.2 | 0.90 | 0.90 | 1.00 | 0.95 | 0.90 | 0.93 | 0.04 |
| S145 | No.2 | 0.90 | 0.90 | 0.90 | 0.95 | 0.95 | 0.92 | 0.03 |
| S134 | No.2 | 0.95 | 1.00 | 0.90 | 0.90 | 0.85 | 0.92 | 0.06 |
| S162 | No.2 | 0.90 | 1.00 | 0.90 | 0.80 | 0.90 | 0.90 | 0.07 |
| S47 | No.5 | 1.00 | 0.80 | 0.80 | 0.90 | 0.90 | 0.88 | 0.08 |
| S35 | No.2 | 0.90 | 0.90 | 0.95 | 0.80 | 0.80 | 0.87 | 0.07 |
| S152 | No.2 | 0.80 | 0.85 | 0.90 | 0.80 | 0.90 | 0.85 | 0.05 |
| S9 | No.2 | 0.90 | 1.10 | 1.00 | 0.60 | 0.60 | 0.84 | 0.23 |
| S142 | No.2 | 0.80 | 0.70 | 0.90 | 0.80 | 0.90 | 0.82 | 0.08 |
| S58 | No.21 | 0.80 | 0.81 | 0.80 | 0.79 | 0.80 | 0.80 | 0.01 |
| S157 | No.2 | 0.70 | 0.70 | 0.80 | 0.90 | 0.90 | 0.80 | 0.10 |
| S3 | No.2 | 0.60 | 0.70 | 0.70 | 0.80 | 0.90 | 0.74 | 0.11 |
| S99 | No.34 | 0.65 | 0.75 | 0.70 | 0.69 | 0.72 | 0.70 | 0.04 |
| S86 | No.13 | 0.70 | 0.68 | 0.68 | 0.71 | 0.72 | 0.70 | 0.02 |
| S12 | No.2 | 0.70 | 0.80 | 0.80 | 0.50 | 0.60 | 0.68 | 0.13 |
| S117 | No.22 | 0.65 | 0.65 | 0.68 | 0.70 | 0.70 | 0.68 | 0.03 |
| S4 | No.2 | 0.50 | 0.70 | 0.70 | 0.50 | 0.60 | 0.60 | 0.10 |
| S69 | No.2 | 0.60 | 0.60 | 0.58 | 0.62 | 0.60 | 0.60 | 0.01 |
| S8 | No.2 | 0.50 | 0.70 | 0.70 | 0.40 | 0.40 | 0.54 | 0.15 |
| S136 | No.2 | 0.50 | 0.55 | 0.45 | 0.55 | 0.50 | 0.51 | 0.04 |
| S98 | No.2 | 0.48 | 0.50 | 0.52 | 0.52 | 0.51 | 0.51 | 0.02 |
| S125 | No.2 | 0.50 | 0.50 | 0.60 | 0.40 | 0.50 | 0.50 | 0.07 |
| S14 | No.2 | 0.50 | 0.40 | 0.55 | 0.50 | 0.50 | 0.49 | 0.05 |
| S6 | No.2 | 0.40 | 0.50 | 0.50 | 0.50 | 0.50 | 0.48 | 0.04 |
| S123 | No.2 | 0.50 | 0.50 | 0.70 | 0.30 | 0.40 | 0.48 | 0.15 |
| S140 | No.2 | 0.50 | 0.50 | 0.50 | 0.30 | 0.40 | 0.44 | 0.09 |
| S128 | No.2 | 0.50 | 0.40 | 0.60 | 0.40 | 0.30 | 0.44 | 0.11 |
| S116 | No.2 | 0.45 | 0.40 | 0.45 | 0.40 | 0.45 | 0.43 | 0.03 |
| S2 | No.2 | 0.30 | 0.50 | 0.50 | 0.40 | 0.40 | 0.42 | 0.08 |
| S156 | No.2 | 0.50 | 0.30 | 0.40 | 0.40 | 0.40 | 0.40 | 0.07 |
| S155 | No.2 | 0.40 | 0.40 | 0.30 | 0.40 | 0.40 | 0.38 | 0.04 |
| S133 | No.2 | 0.50 | 0.30 | 0.40 | 0.30 | 0.30 | 0.36 | 0.09 |
| S158 | No.2 | 0.40 | 0.40 | 0.40 | 0.20 | 0.30 | 0.34 | 0.09 |
| S154 | No.2 | 0.30 | 0.40 | 0.30 | 0.30 | 0.30 | 0.32 | 0.04 |
| S159 | No.2 | 0.30 | 0.30 | 0.40 | 0.20 | 0.30 | 0.30 | 0.07 |
| S135 | No.2 | 0.30 | 0.40 | 0.20 | 0.20 | 0.30 | 0.28 | 0.08 |
| S160 | No.2 | 0.30 | 0.40 | 0.20 | 0.30 | 0.20 | 0.28 | 0.08 |
| S169 | No.2 | 0.20 | 0.30 | 0.40 | 0.20 | 0.20 | 0.26 | 0.09 |
| S36 | No.2 | 0.15 | 0.25 | 0.20 | 0.15 | 0.25 | 0.20 | 0.05 |
| S10 | No.10 | 0.00 | 0.00 | 0.00 | 0.00 | 0.00 | 0.00 | 0.00 |
| S17 | No.11 | 0.00 | 0.00 | 0.00 | 0.00 | 0.00 | 0.00 | 0.00 |
| S18 | No.12 | 0.00 | 0.00 | 0.00 | 0.00 | 0.00 | 0.00 | 0.00 |
| S19 | No.2 | 0.00 | 0.00 | 0.00 | 0.00 | 0.00 | 0.00 | 0.00 |
| S30 | No.2 | 0.00 | 0.00 | 0.00 | 0.00 | 0.00 | 0.00 | 0.00 |
| S33 | No.2 | 0.00 | 0.00 | 0.00 | 0.00 | 0.00 | 0.00 | 0.00 |
| S37 | No.2 | 0.00 | 0.00 | 0.00 | 0.00 | 0.00 | 0.00 | 0.00 |
| S40 | No.28 | 0.00 | 0.00 | 0.00 | 0.00 | 0.00 | 0.00 | 0.00 |
| S45 | No.29 | 0.00 | 0.00 | 0.00 | 0.00 | 0.00 | 0.00 | 0.00 |
| S56 | No.2 | 0.00 | 0.00 | 0.00 | 0.00 | 0.00 | 0.00 | 0.00 |
| S57 | No.18 | 0.00 | 0.00 | 0.00 | 0.00 | 0.00 | 0.00 | 0.00 |
| S60 | No.30 | 0.00 | 0.00 | 0.00 | 0.00 | 0.00 | 0.00 | 0.00 |
| S61 | No.30 | 0.00 | 0.00 | 0.00 | 0.00 | 0.00 | 0.00 | 0.00 |
| S62 | No.2 | 0.00 | 0.00 | 0.00 | 0.00 | 0.00 | 0.00 | 0.00 |
| S63 | No.16 | 0.00 | 0.00 | 0.00 | 0.00 | 0.00 | 0.00 | 0.00 |
| S65 | No.31 | 0.00 | 0.00 | 0.00 | 0.00 | 0.00 | 0.00 | 0.00 |
| S66 | No.2 | 0.00 | 0.00 | 0.00 | 0.00 | 0.00 | 0.00 | 0.00 |
| S71 | No.2 | 0.00 | 0.00 | 0.00 | 0.00 | 0.00 | 0.00 | 0.00 |
| S72 | No.6 | 0.00 | 0.00 | 0.00 | 0.00 | 0.00 | 0.00 | 0.00 |
| S73 | No.6 | 0.00 | 0.00 | 0.00 | 0.00 | 0.00 | 0.00 | 0.00 |
| S74 | No.21 | 0.00 | 0.00 | 0.00 | 0.00 | 0.00 | 0.00 | 0.00 |
| S75 | No.8 | 0.00 | 0.00 | 0.00 | 0.00 | 0.00 | 0.00 | 0.00 |
| S76 | No.8 | 0.00 | 0.00 | 0.00 | 0.00 | 0.00 | 0.00 | 0.00 |
| S77 | No.24 | 0.00 | 0.00 | 0.00 | 0.00 | 0.00 | 0.00 | 0.00 |
| S79 | No.27 | 0.00 | 0.00 | 0.00 | 0.00 | 0.00 | 0.00 | 0.00 |
| S80 | No.30 | 0.00 | 0.00 | 0.00 | 0.00 | 0.00 | 0.00 | 0.00 |
| S81 | No.22 | 0.00 | 0.00 | 0.00 | 0.00 | 0.00 | 0.00 | 0.00 |
| S83 | No.21 | 0.00 | 0.00 | 0.00 | 0.00 | 0.00 | 0.00 | 0.00 |
| S84 | No.14 | 0.00 | 0.00 | 0.00 | 0.00 | 0.00 | 0.00 | 0.00 |
| S87 | No.7 | 0.00 | 0.00 | 0.00 | 0.00 | 0.00 | 0.00 | 0.00 |
| S91 | No.3 | 0.00 | 0.00 | 0.00 | 0.00 | 0.00 | 0.00 | 0.00 |
| S92 | No.33 | 0.00 | 0.00 | 0.00 | 0.00 | 0.00 | 0.00 | 0.00 |
| S93 | No.32 | 0.00 | 0.00 | 0.00 | 0.00 | 0.00 | 0.00 | 0.00 |
| S94 | No.2 | 0.00 | 0.00 | 0.00 | 0.00 | 0.00 | 0.00 | 0.00 |
| S96 | No.21 | 0.00 | 0.00 | 0.00 | 0.00 | 0.00 | 0.00 | 0.00 |
| S97 | No.17 | 0.00 | 0.00 | 0.00 | 0.00 | 0.00 | 0.00 | 0.00 |
| S100 | No.27 | 0.00 | 0.00 | 0.00 | 0.00 | 0.00 | 0.00 | 0.00 |
| S101 | No.2 | 0.00 | 0.00 | 0.00 | 0.00 | 0.00 | 0.00 | 0.00 |
| S102 | No.2 | 0.00 | 0.00 | 0.00 | 0.00 | 0.00 | 0.00 | 0.00 |
| S105 | No.7 | 0.00 | 0.00 | 0.00 | 0.00 | 0.00 | 0.00 | 0.00 |
| S106 | No.3 | 0.00 | 0.00 | 0.00 | 0.00 | 0.00 | 0.00 | 0.00 |
| S107 | No.25 | 0.00 | 0.00 | 0.00 | 0.00 | 0.00 | 0.00 | 0.00 |
| S109 | No.2 | 0.00 | 0.00 | 0.00 | 0.00 | 0.00 | 0.00 | 0.00 |
| S114 | No.30 | 0.00 | 0.00 | 0.00 | 0.00 | 0.00 | 0.00 | 0.00 |
| S115 | No.15 | 0.00 | 0.00 | 0.00 | 0.00 | 0.00 | 0.00 | 0.00 |
| S118 | No.17 | 0.00 | 0.00 | 0.00 | 0.00 | 0.00 | 0.00 | 0.00 |
| S119 | No.7 | 0.00 | 0.00 | 0.00 | 0.00 | 0.00 | 0.00 | 0.00 |
| S120 | No.3 | 0.00 | 0.00 | 0.00 | 0.00 | 0.00 | 0.00 | 0.00 |
| S121 | No.20 | 0.00 | 0.00 | 0.00 | 0.00 | 0.00 | 0.00 | 0.00 |
| S179 | No.24 | 0.00 | 0.00 | 0.00 | 0.00 | 0.00 | 0.00 | 0.00 |
| S180 | No.24 | 0.00 | 0.00 | 0.00 | 0.00 | 0.00 | 0.00 | 0.00 |

**Table S2. *ATG* gene expression identified by RNA-Seq analysis under nitrogen starvation in *Fusarium graminearum***

| Gene Name | Locus | Expression Level | Fold Change(Log_2_)^*^ | FDR |
| --- | --- | --- | --- | --- |
| *ATG1* | FGSG_05547 | Up | 1.63 | 1.74E-13 |
| *ATG2* | FGSG_10283 | *NS* | 0.69 | 1.10E-03 |
| *ATG3* | FGSG_08900 | *NS* | 0.75 | 2.62E-04 |
| *ATG4* | FGSG_17282 | *NS* | 0.28 | 2.17E-01 |
| *ATG5* | FGSG_10053 | Up | 1.86 | 5.96E-24 |
| *ATG6* | FGSG_11805 | *NS* | -0.50 | 1.25E-02 |
| *ATG7* | FGSG_10226 | *NS* | 0.69 | 6.55E-04 |
| *ATG8* | FGSG_10740 | *NS* | 0.98 | 4.62E-06 |
| *ATG9* | FGSG_13660 | Up | 1.16 | 4.28E-08 |
| *ATG11* | FGSG_15734 | *NS* | 0.77 | 3.04E-04 |
| *ATG12* | FGSG_13550 | Up | 2.57 | 4.86E-15 |
| *ATG13* | FGSG_08491 | Down | -1.19 | 2.08E-07 |
| *ATG14* | FGSG_00675 | *NS* | 1.00 | 1.42E-06 |
| *ATG15* | FGSG_02519 | *NS* | -0.70 | 9.50E-04 |
| *ATG16* | FGSG_02566 | *NS* | -0.22 | 4.88E-01 |
| *ATG17* | FGSG_06510 | *NS* | 0.39 | 7.67E-02 |
| *ATG18* | FGSG_04297 | *NS* | 0.29 | 1.58E-01 |
| *ATG20* | FGSG_06950 | *NS* | 0.44 | 4.15E-02 |
| *ATG22* | FGSG_01225 | *NS* | 0.33 | 1.14E-01 |
| *ATG23* | FGSG_02793 | *NS* | -0.68 | 6.58E-04 |
| *ATG24* | FGSG_09157 | *NS* | 0.19 | 4.11E-01 |
| *ATG26* | FGSG_13231 | *NS* | -0.01 | 9.71E-01 |
| *ATG27* | FGSG_01574 | *NS* | -0.35 | 1.18E-01 |
| *ATG28* | FGSG_16842 | *NS* | -0.31 | 1.87E-01 |
| *ATG29* | FGSG_13575 | Up | 1.21 | 7.89E-11 |
| *ATG33* | FGSG_00549 | *NS* | -0.58 | 2.94E-03 |

^*^Fold change Log_2_ ratio≥1 and FDR ≤0.05 were used as the threshold.

**Table S3 List of identified Gcn5-interacting proteins associated with the autophagy process**

| Locus | Protein function | Mascot score* | E-value |
| --- | --- | --- | --- |
| FGSG_02780 | Coatomer subunit beta | 104.76 | 4.98E-10 |
| FGSG_01917 | Protein transport protein SEC23 | 74.74 | 6.88E-07 |
| FGSG_08840 | Coatomer subunit gamma | 73.7 | 1.07E-06 |
| FGSG_08857 | Ras-like C3 botulinum toxin substrate 1 | 61.2 | 1.76E-05 |
| FGSG_02756 | Vacuolar protein sorting-associated protein 35 | 59.18 | 1.52E-05 |
| FGSG_01528 | Protein transport protein Sec61 subunit beta | 57.58 | 4.16E-05 |
| FGSG_04327 | Ras-like protein Rab-11B | 51.99 | 9.74E-05 |
| FGSG_10779 | SEC14 cytosolic factor | 46.88 | 1.72E-04 |
| FGSG_00472 | Serine/threonine-protein kinase Sch9 | 45.8 | 4.56E-04 |
| FGSG_04485 | Arp2/3 complex-mediated actin nucleation | 45.02 | 7.77E-04 |
| FGSG_07939 | Coatomer subunit alpha | 44.91 | 8.43E-04 |
| FGSG_05436 | Coatomer subunit delta | 40.26 | 1.50E-03 |
| FGSG_10856 | Arp2/3 complex-mediated actin nucleation | 39.53 | 1.92E-03 |
| FGSG_00549 | Autophagy-related protein 33 | 39.02 | 4.92E-03 |
| FGSG_06950 | Autophagy-related protein 20 | 38.06 | 3.67E-03 |
| FGSG_09271 | Protein transport protein SEC13 | 37.53 | 1.01E-02 |
| FGSG_10782 | Vacuolar protease A | 29.87 | 9.12E-03 |
| FGSG_10740 | **Autophagy-related protein 8** | **29.53** | **3.03E-02** |
| FGSG_07172 | Vacuolar protein sorting-associated protein 1 | 28.66 | 4.49E-02 |
| FGSG_05844 | Coatomer subunit beta | 26.58 | 3.68E-02 |
| FGSG_00610 | Actin-related protein 2/3 complex subunit | 23.35 | 9.20E-02 |

*Mascot score≥20 was used as the threshold.

**Table S4 Putative histone deacetylases (HDACs) in *F. graminearum***

| *S. cerevisiae* | *F. graminearum* | Percentage positives to *S. c* (%) | e-value |
| --- | --- | --- | --- |
| Hst3 | FGSG_00460 (Hst3) | 49.2 | 2.7E-16 |
| Hos2 | FGSG_01353 (Hdf1) | 68.6 | 1.0E-51 |
| Hda1 | FGSG_04324 (Hda1) | 54.7 | 2.9E-109 |
| Sir2 | FGSG_05505 (Sir2) | 35.9 | 0.0052 |
| Rpd3 | FGSG_00780 (Rpd3) | 74.1 | 0.0 |
| Hos3 | FGSG_05636 (Hos3) | 44.0 | 3.1E-90 |
| Hst2 | FGSG_09218 (Hst2) | 55.7 | 2.9E-51 |
| Hst1 | FGSG_13552 (Hst1-1) | 47.5 | 1.1E-7 |
|  | FGSG_17227 (Hst1-2) | 54.6 | 2.1E-48 |
| Hst4 | FGSG_16002 (Hst4) | 43.4 | 2.6E-19 |

**Table S5. PCR primers used in this study**

| **Primers** | **Sequence (5’ to 3’)** | **Relevant characteristics** |
| --- | --- | --- |
| HPH-F | GGAGGTCAACACATCAATGCCTATT | PCR primers to amplify fragment of HPH |
| HPH-R | CTACTCTATTCCTTTGCCCT |  |
| G418-F | GGAGGTCAACACATCAATGCT | PCR primers to amplify fragment of G418 |
| G418-R | TCAGAAGAACTCGTCAAGAAG |  |
| NTC1-F | ACTAGTGATATTGAAGGAGC | PCR primers to amplify fragment of NTC1 |
| NTC1-R | AGGCCTGATGCTTTGGTTTAG |  |
| Atg8-UP-F | TGGCATCGATGACTTCTCTAC | PCR primers to amplify upstream fragment of *ATG8* |
| Atg8-UP-R | CAAAATAGGCATTGATGTGTTGACCTCCGACGGTGATGGTTGTTGTGGA |  |
| Atg8-DOWN-F | CTCGTCCGAGGGCAAAGGAATAGAGTAGGGCCGGTGATGCAGGATCGGT | PCR primers to amplify downstream fragment of *ATG8* |
| Atg8-DOWN-R | CATTGAAGGTAGGGTTCCATGC |  |
| Atg8-ID-F | CACCTACTTATTCTGCCTGAC | PCR primers for identification of *ATG8* deletion transformants |
| Atg8-ID-R | GGTTGGTGGTTACAATTTGTC |  |
| Atg3-UP-F | CGAACCATCACTGTCATGGAC | PCR primers to amplify upstream fragment of *ATG3* |
| Atg3-UP-R | CAAAATAGGCATTGATGTGTTGACCTCCGCTTCGAGATTGATAACTTC |  |
| Atg3-DOWN-F | CTCGTCCGAGGGCAAAGGAATAGAGTAGCAGGAAGCCGAGATTGATG | PCR primers to amplify downstream fragment of *ATG3* |
| Atg3-DOWN-R | GTGTGTAACCCAAGCCATCC |  |
| Atg3-ID-F | GCGTGACGGAAGGACGAGG | PCR primers for identification of *ATG3* deletion transformants |
| Atg3-ID-R | CCATGGTGAAATCGTGCTCG |  |
| Atg7-UP-F | GCTCTGTTCTTCTAGAAGGTC | PCR primers to amplify upstream fragment of *ATG7* |
| Atg7-UP-R | CAAAATAGGCATTGATGTGTTGACCTCCGTGACGGTAGAGCTGCTGCTC |  |
| Atg7-DOWN-F | CTCGTCCGAGGGCAAAGGAATAGAGTAGAAGACTGTAAAGGACATTTG | PCR primers to amplify downstream fragment of *ATG7* |
| Atg7-DOWN-R | GCTCTATTCTTCCGTTAGTC |  |
| Atg7-ID-F | GGCCTCGAACTAAGAATAGC | PCR primers for identification of *ATG7* deletion transformants |
| Atg7-ID-R | GCCGATAATAATTGGATGCC |  |
| Gcn5-UP-F | TTTGGACATGAACTCTGGGTG | PCR primers to amplify upstream fragment of *GCN5* |
| Gcn5-UP-R | CAAAATAGGCATTGATGTGTTGACCTCCAACCCAGGTTGTTGGGACTT |  |
| Gcn5-DOWN-F | CTCGTCCGAGGGCAAAGGAATAGAGTAGACAAGAAGGTCTGGATGGGTT | PCR primers to amplify downstream fragment of *GCN5* |
| Gcn5-DOWN-R | GATGCCTGGGCATGATCTTTA |  |
| Gcn5-ID-F | GCATCGTGGCCATAGACTAAT | PCR primers for identification of *GCN5* deletion transformants |
| Gcn5-ID-R | TTCGGAGGAGCATGGATGTT |  |
| Esa1-UP-F | GGTGCCTCTGCCTTGTCTGT | PCR primers to amplify upstream fragment of *ESA1* |
| Esa1-UP-R | CAAAATAGGCATTGATGTGTTGACCTCCGTTTGTGCGCTTGTCGTAAT |  |
| Esa1-DOWN-F | CTCGTCCGAGGGCAAAGGAATAGAGTAGGTAGCCACAGATGATGAGAG | PCR primers to amplify downstream fragment of *ESA1* |
| Esa1-DOWN-R | TGGAAAGAGATGCAGGTGAT |  |
| Esa1-ID-F | GCGATGAACAAGACGTGACC | PCR primers for identification of *ESA1* deletion transformants |
| Esa1-ID-R | ACAAACAAAAGCCTCTATCG |  |
| Hat2-UP-F | ACCCATCAGATAAATGGCGA | PCR primers to amplify upstream fragment of *HAT2* |
| Hat2-UP-R | CAAAATAGGCATTGATGTGTTGACCTCCCCTTGGAAATGGATTATGACG |  |
| Hat2-DOWN-F | CTCGTCCGAGGGCAAAGGAATAGAGTAGAATTACGCGCGATCTACTCTG | PCR primers to amplify downstream fragment of *HAT2* |
| Hat2-DOWN-R | TGCTGGTATCACTGTTGAGGA |  |
| Hat2-ID-F | TTAATTGAAACGCGCGGA | PCR primers for identification of *HAT2* deletion transformants |
| Hat2-ID-R | GACAGGCCTTTTAATGGCTT |  |
| Hpa2-UP-F | CTCGCGCATATACGATTCTGA | PCR primers to amplify upstream fragment of *HPA2* |
| Hpa2-UP-R | CAAAATAGGCATTGATGTGTTGACCTCCTTGGGGAGATAGCGATGTCAT |  |
| Hpa2-DOWN-F | CTCGTCCGAGGGCAAAGGAATAGAGTAGCCCATAGGTCTGATGGAAACT | PCR primers to amplify downstream fragment of *HPA2* |
| Hpa2-DOWN-R | GTAGTGAAGTGCCATGACCTG |  |
| Hpa2-ID-F | CACCTTGGGATCTGTACCAGT | PCR primers for identification *HPA2* deletion transformants |
| Hpa2-ID-R | TTGATCGTCGCTGACAAAGA |  |
| Elp3-UP-F | TTTGTTTAACCCCGCAGTGA | PCR primers to amplify upstream fragment of *ELP3* |
| Elp3-UP-R | CAAAATAGGCATTGATGTGTTGACCTCCATGGGGACTGATTTTGCGAT |  |
| Elp3-DOWN-F | CTCGTCCGAGGGCAAAGGAATAGAGTAGTGCAGAGTACAGGGTAACGAA | PCR primers to amplify downstream fragment of *ELP3* |
| Elp3-DOWN-R | GGCATCTAAAGGTGCTGCTAA |  |
| Elp3-ID-F | CGTGACCGAGTTATTGACCTT | PCR primers for identification of *ELP3* deletion transformants |
| Elp3-ID-R | TTCCAAGATCTGAGGCATCA |  |
| Spt10-UP-F | GTTGTCTGCGGACAAATGGTT | PCR primers to amplify upstream fragment of *SPT10* |
| Spt10-UP-R | CAAAATAGGCATTGATGTGTTGACCTCCTTTCTTGGAGCGTACCCTTT |  |
| Spt10-DOWN-F | CTCGTCCGAGGGCAAAGGAATAGAGTAGCTTGTCGTCGTTTTGTTGGA | PCR primers to amplify downstream fragment of *SPT10* |
| Spt10-DOWN-R | AACTTCTTCGAGAACTGCCCT |  |
| Spt10-ID-F | ACTGCGAGCATAATTCGTGA | PCR primers for identification of *SPT10* deletion transformants |
| Spt10-ID-R | AGCCCCAACTTGAACGTTTA |  |
| Sas2-UP-F | GAGCCGACCAAACCACAGAT | PCR primers to amplify upstream fragment of *SAS2* |
| Sas2-UP-R | CAAAATAGGCATTGATGTGTTGACCTCCTGTGCGACCCTCTTCTCTTG |  |
| Sas2-DOWN-F | CTCGTCCGAGGGCAAAGGAATAGAGTAGAAGCATAGACAGTTTAGACG | PCR primers to amplify downstream fragment of *SAS2* |
| Sas2-DOWN-R | GATTTTCAGCCTTCCTTGTT |  |
| Sas2-ID-F | CCATCCTATCGCAAATCAAT | PCR primers for identification of *SAS2* deletion transformants |
| Sas2-ID-R | GCAGGGGTATCACATCAGAG |  |
| Rtt109-UP-F | TTGAGACTGCCCTTGCAGAA | PCR primers to amplify upstream fragment of *RTT109* |
| Rtt109-UP-R | CAAAATAGGCATTGATGTGTTGACCTCCTGCAGGGGTACCTACTAAGGT |  |
| Rtt109-DOWN-F | CTCGTCCGAGGGCAAAGGAATAGAGTAGTTTGGGTAATGATTGGTTTGA | PCR primers to amplify downstream fragment of *RTT109* |
| Rtt109-DOWN-R | TTTTGGGTGAACGAGGTGAGT |  |
| Rtt109-ID-F | ATGAAGTACCTTGAACCGTCC | PCR primers for identification of *RTT109* deletion transformants |
| Rtt109-ID-R | TCGTGTACTTTTGACGTGCCA |  |
| Hst4-UP-F | TGTCACCAAACGCCAATGTC | PCR primers to amplify upstream fragment of *HST4* |
| Hst4-UP-R | CAAAATAGGCATTGATGTGTTGACCTCCCTCGTCGGGAAATCAACAAT |  |
| Hst4-DOWN-F | CTCGTCCGAGGGCAAAGGAATAGAGTAGTATCTTGTCATCTGTTGCCT | PCR primers to amplify downstream fragment of *HST4* |
| Hst4-DOWN-R | ATGGCGACGAAACAAACTAT |  |
| Hst4-ID-F | ATTAGAGTGAGTAGGCAGGC | PCR primers for identification of *HST4* deletion transformants |
| Hst4-ID-R | GCAAACCTCCCCGATAATGT |  |
| Hdf1-UP-F | TGATAGGTTCTTTTTGGCTC | PCR primers to amplify upstream fragment of *HDF1* |
| Hdf1-UP-R | CAAAATAGGCATTGATGTGTTGACCTCCACAGGAGTATGAGGAGTGAT |  |
| Hdf1-DOWN-F | CTCGTCCGAGGGCAAAGGAATAGAGTAGTTATGTGGATGATGGTTCTG | PCR primers to amplify downstream fragment of *HDF1* |
| Hdf1-DOWN-R | ATTGAGATACGAGTGTTGGC |  |
| Hdf1-ID-F | TTCTCTAACACACTGGGCTG | PCR primers for identification of *HDF1* deletion transformants |
| Hdf1-ID-R | CAGCCGTCATTCAGGATAAG |  |
| Hda2-UP-F | AAACGGTAGTAGTGAGTGAT | PCR primers to amplify upstream fragment of *HDA2* |
| Hda2-UP-R | CAAAATAGGCATTGATGTGTTGACCTCCTCTAATGACCCCAGGAGTTG |  |
| Hda2-DOWN-F | CTCGTCCGAGGGCAAAGGAATAGAGTAGTAGTATGAGGAAGACGGTGT | PCR primers to amplify downstream fragment of *HDA2* |
| Hda2-DOWN-R | AATCTTGAGTCGGGTTTGGG |  |
| Hda2-ID-F | TTCCTTTCCCCTCCACCTGT | PCR primers for identification of *HDA2* deletion transformants |
| Hda2-ID-R | AAACTCCTTCATCTCCATTC |  |
| Sir2-UP-F | GAGAAAGAGAGAAAGAAACG | PCR primers to amplify upstream fragment of *SIR2* |
| Sir2-UP-R | CAAAATAGGCATTGATGTGTTGACCTCCGTTTATTCCAGAGTCGTAGT |  |
| Sir2-DOWN-F | CTCGTCCGAGGGCAAAGGAATAGAGTAG CAGGGGCGAGGATAAGTTTT | PCR primers to amplify downstream fragment of *SIR2* |
| Sir2-DOWN-R | AATCTTTGTCACTCTTTGGG |  |
| Sir2-ID-F | TTCCACCAATCCTCCACGAG | PCR primers for identification of *SIR2* deletion transformants |
| Sir2-ID-R | TTGAAGATGCCAGGATTTGC |  |
| Rpd3-UP-F | TAACGCACCTTCCTGTCTGT | PCR primers to amplify upstream fragment of *RPD3* |
| Rpd3-UP-R | CAAAATAGGCATTGATGTGTTGACCTCCGAATCTCGGGAAATGGGTGG |  |
| Rpd3-DOWN-F | CTCGTCCGAGGGCAAAGGAATAGAGTAGGCCAGGGAATAAGCAACTCG | PCR primers to amplify downstream fragment of *RPD3* |
| Rpd3-DOWN-R | GCTAACTGCCGCTGAAAGAC |  |
| Rpd3-ID-F | GTTTTCGCTGTTCCACGCAT | PCR primers for identification of *RPD3* deletion transformants |
| Rpd3-ID-R | ACCACATCTTATCGTCGCTT |  |
| Hos3-UP-F | GCCACTCACTACCAACCTAT | PCR primers to amplify upstream fragment of *HOS3* |
| Hos3-UP-R | CAAAATAGGCATTGATGTGTTGACCTCCTATCAGACCCGTTTCGTAGC |  |
| Hos3-DOWN-F | CTCGTCCGAGGGCAAAGGAATAGAGTAGGCCAACCTCCCACATTCTGC | PCR primers to amplify downstream fragment of *HOS3* |
| Hos3-DOWN-R | CACAGGGAAAAAGCAGCCAG |  |
| Hos3-ID-F | TATTCCCAAGGCGATTAGAG | PCR primers for identification of *HOS3* deletion transformants |
| Hos3-ID-R | TCACAAAACAGGCAGCCAGC |  |
| Hst2-UP-F | ACTGTCCAAAAGATTGTGTC | PCR primers to amplify upstream fragment of *HST2* |
| Hst2-UP-R | CAAAATAGGCATTGATGTGTTGACCTCCTATTTTGGCGACGATGGGAG |  |
| Hst2-DOWN-F | CTCGTCCGAGGGCAAAGGAATAGAGTAGCTACGAATTACTGTCATCAC | PCR primers to amplify downstream fragment of *HST2* |
| Hst2-DOWN-R | CTGTAGCGTAAGGAACTGAC |  |
| Hst2-ID-F | CGCCAAAGTGAAAAATGATG | PCR primers for identification of *HST2* deletion transformants |
| Hst2-ID-R | GACCTCAGCCAACACGAATC |  |
| Hst1-1-UP-F | GGGAAGAAAACACGAGGGAT | PCR primers to amplify upstream fragment of *HST1-1* |
| Hst1-1-UP-R | CAAAATAGGCATTGATGTGTTGACCTCCGCAGGGGTTAGAAGTAGACT |  |
| Hst1-1-Down-F | CTCGTCCGAGGGCAAAGGAATAGAGTAGGAGATTTTGCCTTTGGTCAG | PCR primers to amplify downstream fragment of *HST1-1* |
| Hst1-1-Down-R | AGCCTTCCGTAGTTTCTGAT |  |
| Hst1-1-ID-F | ATTCCCTTACAACAGCCATC | PCR primers for identification of *HST1-1* deletion transformants |
| Hst1-1-ID-R | CTTCCTCAGTTCCCCTCCTT |  |
| Hst3-UP-F | AAGGGTTGGGACGCCATCAC | PCR primers to amplify upstream fragment of *HST3* |
| Hst3-UP-R | CAAAATAGGCATTGATGTGTTGACCTCCAAGAAAACGAAGAGGGAAAG |  |
| Hst3-DOWN-F | CTCGTCCGAGGGCAAAGGAATAGAGTAGACGACGGATGACAAACCACT | PCR primers to amplify downstream fragment of *HST3* |
| Hst3-DOWN-R | CGAGGGGAGCGTTGATGATC |  |
| Hst3-ID-F | CACCATTCGTTACACTTCAC | PCR primers for identification of *HST3* deletion transformants |
| Hst3-ID-R | ATACTACTTTGTTCCCTGTC |  |
| Hst1-2-UP-F | CACTCTGGGTTTTAGGATAG | PCR primers to amplify upstream fragment of *HST1-2* |
| Hst1-2-UP-R | CAAAATAGGCATTGATGTGTTGACCTCCGATGAACGGGGATTTAGATG |  |
| Hst1-2-DOWN-F | CTCGTCCGAGGGCAAAGGAATAGAGTAGTTCATTTCGCATTGCTCTTG | PCR primers to amplify downstream fragment of *HST1-2* |
| Hst1-2-DOWN-R | GTTTCGGGTTTCCTGGGTAG |  |
| Hst1-2-ID-F | TCTTTACCTACTTCCTTACC | PCR primers for identification of *HST1-2* deletion transformants |
| Hst1-2-ID-R | AGATACCATTTTACTCCGTC |  |
| Atg8-C-F1 | GCTCCTTTATATGAGAAGCAGAT | Upstream of *ATG8* for in locus complementation |
| Atg8-C-R1 | CAAAATAGCATTGATGTGTTGACCTCCAGGGTCGAGAATGAGAGTTTG |  |
| Atg8-C-F2 | CAAACTCTCATTCTCGACCCTGGAGGTCAACACATCAATGCTATTTTG | TrpC-neo fragment of *ATG8* for *in locus* complementation |
| Atg8-C-R2 | ATTTGAGTACCCAATTCGCCCTATAGTGAGTTCAGAAGAACTCGTCAAGAAGGCGA |  |
| Atg8-C-F3 | TCGCCTTCTTGACGAGTTCTTCTGAACTCACTATAGGGCGAATTGGGTACTCAAAT | RP27-GFP-Atg8 fragment of *ATG8* for *in locus* complementation |
| Atg8-C-R3 | AAGATAGAACCGATCCTGCATCACCGGCCTTACGCTTCGCCAAAAGTGTTCTCG |  |
| Atg8-C-F4 | CGAGAACACTTTTGGCGAAGCGTAAGGCCGGTGATGCAGGATCGGTTCTATCTT | Downstream of *ATG8* for *in locus* complementation |
| Atg8-C-R4 | TTCCCAAACAAAGCGCTGTTCTTCGAAAAC |  |
| RP27-F | ACTCACTATAGGGCGAATTGGGTACTCAAATTGGTT | PCR primers to amplify RP27 promoter for N-terminal GFP fused complementation |
| RP27-R | TTTGAAGATTGGGTTCCTACGAAA |  |
| GFP-F | TTTCGTAGGAACCCAATCTTCAAAATGGTGAGCAAGGGCGAGGAG | PCR primers to amplify GFP for N-terminal GFP fused complementation |
| GFP-R | CTTGTACAGCTCGTCCATGCCGAGAG |  |
| Atg8-K13R-F | CTCTCGGCATGGACGAGCTGTACAAGATGCGCAGCAAATTCAAGGACGAGCACCCCTTCGAGAGGCGCAAGGCT | PCR primers to amplify fragment of K13R-mutated-Atg8 for N-terminal GFP fused complementation |
| Atg8-K13R-R | TTCCCAAACAAAGCGCTGTTCTTCGAAAAC |  |
| Atg8-K13Q-F | CTCTCGGCATGGACGAGCTGTACAAGATGCGCAGCAAATTCAAGGACGAGCACCCCTTCGAGCAGCGCAAGGCT | PCR primers to amplify fragment of K13Q-mutated-Atg8 for N-terminal GFP fused complementation |
| Atg8-K13Q-R | ATGCGCAGCAAATTCAAGGACGAGCACCCCTTCGAGCAGCGCAAGGCT |  |
| Atg8-K38R-F1 | AAGTCAGGTCAAGTCTGCGCT | PCR primers to amplify A-fragment of K38R-mutated-Atg8 for N-terminal GFP fused complementation |
| Atg8-K38R-R1 | AATATCACTTCTCTCGACCTTCTCGCAGATAAC |  |
| Atg8-K38R-F2 | GTTATCTGCGAGAAGGTCGAGAGAAGTGATATT | Forward primer to amplify B fragment of K38R-mutated-Atg8 for N-terminal GFP fused complementation |
| Atg8-K38Q-F1 | AAGTCAGGTCAAGTCTGCGCT | PCR primers to amplify A-fragment of K38Q-mutated-Atg8 for N-terminal GFP fused complementation |
| Atg8-K38Q-R1 | AATATCACTTTCCTCGACCTTCTCGCAGATAAC |  |
| Atg8-K38Q-F2 | GTTATCTGCGAGAAGGTCGAGGAAAGTGATATT | Forward primer to amplify B fragment of K38R-mutated-Atg8 for N-terminal GFP fused complementation |
| Atg8-K38RQ-R2 | TCACATGAAGGGCCAGACATTGTCAT | Reverse primer to amplify B fragment of K38R/Q-mutated-Atg8 for N-terminal GFP fused complementation |
| ID-Atg8-F | ATGCGCAGCAAATTCAAGGA | PCR primers for identification site-mutated-FgAtg8 ransformants |
| ID-Atg8-R | TTACGCTTCGCCAAAAGTGTTCTC |  |
| ATG8-CK38R/Q-R1 | GCTCCTTCAATATCACTAGTAGGGTCGAGAATGAGAGTTTG | Reverse primer with Nat1 adaptor to amplify B fragment of K38R/Q-mutated-Atg8 for N-terminal GFP fused complementation |
| Gcn5-C-F1 | TTTGGACATGAACTCTGGGTG | Upstream fragment of *GCN5* for *in locus* complementation |
| Gcn5-C-R1 | AGGACTTACTTTCGTCTGACATGATTGGTGCGGGCTCAACCCAGGTTG |  |
| Gcn5-C-F2 | CAACCTGGGTTGAGCCCGCACCAATCATGTCAGACGAAAGTAAGTCCT | PCR primers to amplify fragment of *GCN5* for C-terminal GFP fused Gcn5 complementation |
| Gcn5-C-R2 | AACAGCTCCTCGCCCTTGCTCACTTTCTCAGGCTCCAGGTGAGACCA |  |
| Gcn5-C-F3 | TGGTCTCACCTGGAGCCTGAGAAAGTGAGCAAGGGCGAGGAGCTGTT | PCR primers to amplify GFP for C-terminal GFP fused Gcn5 complementation |
| Gcn5-C-R3 | CAAAATAGCATTGATGTGTTGACCTCCTTACTTGTACAGCTCGTCCATGCCGAGA |  |
| Gcn5-C-F4 | TCTCGGCATGGACGAGCTGTACAAGTAAGGAGGTCAACACATCAATGCTATTTTG | PCR primers for trpC-neo of *GCN5* for in locus complementation |
| Gcn5-C-R4 | AGTTTTTTGACTGGATGAGATCTTCAGAAGAACTCGTCAAGAAGGCGAT |  |
| Gcn5-C-F5 | ATCGCCTTCTTGACGAGTTCTTCTGAAGATCTCATCCAGTCAAAAAACT | Downstream fragment of *GCN5* for *in locus* complementation |
| Gcn5-C-R5 | GTGACCTGATGATAAGACATG |  |
| Gcn5-mChC-R2 | TCCTCGCCCTTGCTCACCATTTTCTCAGGCTCCAGGTGA | Reverse primer of A-fragement Gcn5-mCh *in locus* complementation |
| Gcn5-mChC-F3 | ATGGTGAGCAAGGGCGAGGA | PCR primers to amplify mCherry fragement for Gcn5-mCh *in locus* complementation |
| Gcn5-mChC-R3 | TTACTTGTACAGCTCGTCCATGC |  |
| Gcn5-mChC-F4 | GCATGGACGAGCTGTACAAGTAAAGATCTCATCCAGTCAAAAAA | Forward primer of B-fragement for Gcn5-mCh in locus complementation |
| mCh-E130Q-R1 | AAAGGACGCCGTTTCGCCCAGATTGTCTTCTGTG | Reverse primer of A-fragement Gcn5-mCh-E130Q site-direct mutation |
| mCh-E130Q-F2 | CACAGAAGACAATCTGGGCGAAACGGCGTCCTTT | Forward primer of B-fragement Gcn5-mCh-E130Q site-direct mutation |
| mCh-E130Q-R2 | TCCTCGCCCTTGCTCACCATTTTCTCAGGCTCCAGGTGAGA |  |
| mCh-E130Q-F3 | ATGGTGAGCAAGGGCGAGGA | mCherry fragement for Gcn5-mCh-E130Q site-direct mutation |
| mCh-E130Q-R3 | TTACTTGTACAGCTCGTCCATGC |  |
| mCh-E130Q-F4 | GCATGGACGAGCTGTACAAGTAAACTAGTGATATTGAAGGAGC | Nat1 fragement for Gcn5-mCh-E130Q site-direct mutation |
| mCh-E130Q-R4 | AGGCCTGATGCTTTGGTTTAG |  |
| mCh-E130Q-F5 | AGGGCAAAGGAATAGAGTAGAGATCTCATCCAGTCAAAAAA | Reverse primer with NAT1 adaptor for downstream of *GCN5* for in locus complementation |
| ID-Gcn5-F | ATGTCAGACGAAAGTAAGTCCTAC | PCR primers for identification site-mutated-FgAtg8 transformants |
| ID-Gcn5-R | TTTCTCAGGCTCCAGGTGAGACCA |  |
| gPDA-F | AAGTCCCAACAACCTGGGTT | PCR primers to amplify *gpda* promoter for *GCN5* *in locus* overexpression |
| gPDA-R | GGTGATGTCTGCTCAAGCGGGGTA |  |
| gPDA-G5-R1 | AACCCAGGTTGTTGGGACTTAACCCAGGTTGTTGGGACTT | Reverse primer 1 with *gpda* adaptor for *GCN5* *in locus* overexpression |
| gPDA-G5-F2 | ATGTCAGACGAAAGTAAGTCCTACTACCCCGCTTGAGCAGACATCACC | Forward primer 2 with *gpda* adaptor for *GCN5* *in locus* overexpression |
| RFP-ID-R | GGAGCCGTACTGGAACTGA | Universal reverse primer for identification of C-terminal RFP fused complementation |
| GFP-ID-R | CGTCGTCCTTGAAGAAGATG | Universal reverse primer for identification of C-terminal GFP fused complementation |
| C-FLAG-ID-R | AATTGAGAAACAGTTAGCATGC | Universal reverse primer for identification of C-terminal Flag fused complementation |
| N-GFP-ID-F | TATATCATGGCCGACAAGCA | Forward primer for identification of N-terminal GFP fused complementation |
| N-FLAG-ID-F | CTCGAAGAGTCAGTCTCCCTT | Forward primer for identification of N-terminal Flag fused complementation |
| RT-GCN5-F | GCCTTCATCTCCTACACCAT | PCR primers of*GCN5* for qRT-PCR |
| RT-GCN5-R | AGGATGATGAGTGCTTCGCG |  |
| RT-HDF1-F | AAAAACCCTCCGAAACGCCTG | PCR primers of *HDF1* for qRT-PCR |
| RT-HDF1-R | TTCTCGTGCTTTGCGGATCT |  |
| RT-ACTIN-F | ATCCACGTCACCACTTTCAA | PCR primers of *ACTIN* for qRT-PCR |
| RT-ACTIN-R | TGCTTGGAGATCCACTTTG |  |
| ATtg8-PET22-F | GTTTAACTTTAAGAAGGAGATATACATATGATGCGCAGCAAATTCAAGGACGA | PCR primers to amplify the fragment of Atg8 for construction of pET22b-Atg8 |
| Atg8-PET22-R | ATCTCAGTGGTGGTGGTGGTGGTGCTCGAGCGCTTCGCCAAAAGTGTTCTCG |  |
| Gcn5-PET22-F | taagaaggagatatacatatgATGTCAGACGAAAACGGCAAG | PCR primers to amplify the fragment of Gcn5 for construction of pET22b-Gcn5 |
| Gcn5-PET22-R | gtggtggtggtggtgctcgagTTTCTCAGGCTCCAGGTGAGAC |  |
| Gcn5-pGEX43-F | tccccgaattcccgggtcgacATGTCAGACGAAAACGGCAAG | PCR primers to amplify the fragment of Gcn5 for construction of pGEX4T-3-Gcn5 |
| Gcn5-pGEX43-R | gatgcggccgctcgagtcgacCTATTTCTCAGGCTCCAGGTGAG |  |
| Hdf1-pGEX43-F | tccccgaattcccgggtcgacATGGATATAGACTCCTACAGGTAT | PCR primers to amplify the fragment of Hdf1 for construction of pGEX4T-3-Hdf1 |
| Hdf1-pGEX43-R | gatgcggccgctcgagtcgacCTAGAGTTGCATTGCAGTTCCTA |  |
| ID-PET22-F | AGATCTCGATCCCGCGAAATTAATAC | PCR primers for identification pET22b-FgX transformants |
| ID-PET22-R | AAACCCCTCAAGACCCGTTTAGAGGC |  |
| ID-pGEX43-F | CTGGTTCCGCGTGGATCCCCGAAT | PCR primers for identification pGEX4T-3-FgX transformants |
| ID-pGEX43-R | ATTTGGAGTACGCCCGGATCTTTGCTTGG |  |
| Atg8-AD-F | gtaccagattacgctcatatgCGCGCTGCTGCCTCTCAA | PCR primers to amplify the fragment of Atg8 for construction of pGADT7-Atg8 |
| Atg8-AD-R | actggcctccatggccatatgGTGGTTGGTGGTTACAATTTGTCT |  |
| Atg8-BD-F | gtaccagattacgctcatatgTCATCGGAAGAGAGTAGTAACAAAGG | PCR primers to amplify the fragment of Atg8 for construction of pGBKT7-Atg8 |
| Atg8-BD-R | actggcctccatggccatatgAAGGGGTTATGCTAGTTATGCGG |  |
| Atg3-AD-F | gccatggaggccagtgaattcATGAATTTCCTCTACTCAACAGTTAACAC | PCR primers to amplify the fragment of Atg3 for construction of pGADT7-Atg3 |
| Atg3-AD-R | atgcccacccgggtggaattcCTAGACGCCCATGGTGAAATCG |  |
| Atg3-BD-F | atggccatggaggccgaattcATGAATTTCCTCTACTCAACAGTTAACAC | PCR primers to amplify the fragment of Atg3 for construction of pGBKT7-Atg3 |
| Atg3-BD-R | tcgacggatccccgggaattcCTAGACGCCCATGGTGAAATCG |  |
| Atg7-AD-F | gccatggaggccagtgaattcATGGCGAAGCCTTTGCAGT | PCR primers to amplify the fragment of Atg7 for construction of pGADT7-Atg3 |
| Atg7-AD-R | atgcccacccgggtggaattcCTAAATAAGTTCTCCTTCACCATCGT |  |
| Atg7-BD-F | atggccatggaggccgaattcATGGCGAAGCCTTTGCAGT | PCR primers to amplify the fragment of Atg7 for construction of pGBKT7-Atg7 |
| Atg7-BD-R | tcgacggatccccgggaattcCTAAATAAGTTCTCCTTCACCATCGT |  |
| Gcn5-AD-F | gccatggaggccagtgaattcATGTCAGACGAAAACGGCAAG | PCR primers to amplify the fragment of Gcn5 for construction of pGADT7-Gcn5 |
| Gcn5-AD-R | atgcccacccgggtggaattcCTATTTCTCAGGCTCCAGGTGAG |  |
| Gcn5-BD-F | atggccatggaggccgaattcATGTCAGACGAAAACGGCAAG | PCR primers to amplify the fragment of Gcn5 for construction of pGBKT7-Gcn5 |
| Gcn5-BD-R | tcgacggatccccgggaattcCTATTTCTCAGGCTCCAGGTGAG |  |
| Hdf1-AD-F | gccatggaggccagtgaattcATGGATATAGACTCCTACAGGTATCGG | PCR primers to amplify the fragment of Hdf1 for construction of pGADT7-Hdf1 |
| Hdf1-AD-R | atgcccacccgggtggaattcCTAGAGTTGCATTGCAGTTCCTACC |  |
| Hdf1-BD-F | atggccatggaggccgaattcATGGATATAGACTCCTACAGGTATCGG | PCR primers to amplify the fragment of Hdf1 for construction of pGBKT7-Hdf1 |
| Hdf1-BD-R | tcgacggatccccgggaattcCTAGAGTTGCATTGCAGTTCCTACC |  |
| ID-AD-F | TACCACTACAATGGATGATG | PCR primers for identification pGADT7-X transformants |
| ID-AD-R | CAGTATCTACGATTCATCTGC |  |
| ID-BD-F | TAATACGACTCACTATAGGG | PCR primers for identification pGBKT7-X transformants |
| ID-BD-R | TAAGAGTCACTTTAAAATTTGTAT |  |
| C-H1-mCh-F1 | CCTGTCCCGTCGCGAAGCTGT | PCR primers to amplify the first fragment of H1 for H1-mCherry label |
| C-H1-mCh-R1 | CGCCTTGGCAGCAGCAGCA |  |
| C-H1-mCh-F2 | TGCTGCTGCTGCCAAGGCGATGGTGAGCAAGGGCGAGGA | PCR primers to amplify the second fragment of H1 for H1-mCherry label |
| C-H1-mCh-R2 | TTACTTGTACAGCTCGTCCATGC |  |
| C-H1-mCh-F3 | GCATGGACGAGCTGTACAAGTAAACTAGTGATATTGAAGGAGC | PCR primers to amplify the third fragment of H1 for H1-mCherry label |
| C-H1-mCh-R3 | AGGCCTGATGCTTTGGTTTAG |  |
