# Supplementary material for: Post-translational regulation of autophagy is involved in intra-microbiome suppression of fungal pathogens
Source: Microbiome. 2021 Jun 6;9:131. doi: 10.1186/s40168-021-01077-y (PMC8182927; doi:10.1186/s40168-021-01077-y)
Supplement: Supplementary file 3 — Additional file 2: Figure S1. Rapamycin or nitrogen starvation effectively induce autophagy and reduce global acetylome in F. graminearum. (a) GFP-Atg8 localization patterns in the wild type strain PH-1 grown in completed medium (CM). Bar = 10 μm. Vacuoles were stained with the CMAC dye. (b) Mass spectrometry data of rapamycin purified from S89 supernatant. The rapamycin producing model strain S. hygroscopicus NRRL5491 and pure rapamycin were used as positive control samples. (c) Autophagy flux of the fungus upon rapamycin or MM-N treatment. GFP-Atg8 labeled PH-1 was grown in CM before rapamycin or MM-N treatment. (d) Representative lysine acetylome profiling of F. graminearum upon nitrogen starvation. Total proteins were extracted from fresh mycelia and analyzed by immunoblot using an anti-acetyl-lysine antibody. Hybridization with an anti-GAPDH antibody served as an internal control. The asterisk indicates acetylated histones. Figure S2. 3-MA treatment inhibits autophagy in F. graminearum. (a) GFP-Atg8 labeled PH-1 was grown in complete medium (CM) and treated with rapamycin in the presence/absence of 3-MA. Autophagic bodies in vacuoles were stabilized by the inhibitor bafilomycin A1. Vacuoles were stained with the CMAC dye. Bar=10 μm. (b) Quantification of the GFP-Atg8 puncta in (a). The data are presented as mean ± s. d., n = 30 compartments. **, P < 0.01. (c) Autophagy flux of the WT strain in the presence/absence of 3-MA upon rapamycin treatment. Figure S3. Deletion and in locus overexpression of GCN5, and autophagy flux of the wild-type, Δgcn5 or OE-GCN5 upon rapamycin treatment. (a) Diagram of GCN5 deletion and in locus overexpression. F1 marked the forward primer for identification of genetic transformants of Gcn5. R1 and R2 are the designed reverse primers for identification. (b) Identification of Δgcn5 and OE-GCN5 strains by PCR assay. M: marker. (c) Relative mRNA level of GCN5 in the wild-type PH-1 (WT) or OE-GCN5 strain. Total RNA extracted from PH-1 or [file 40168_2021_1077_MOESM3_ESM.pdf]

Figure S1

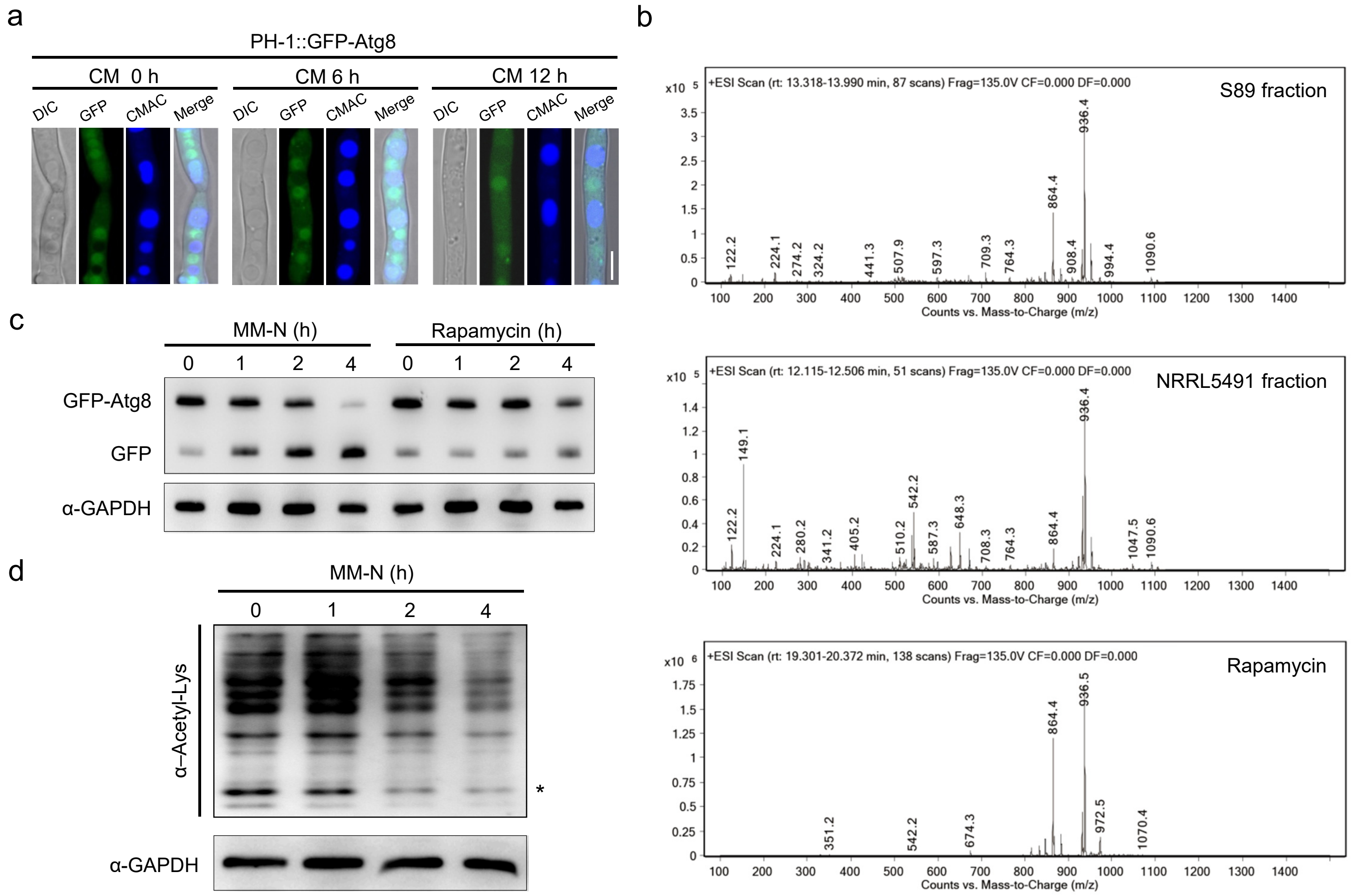

Fig. S1 Rapamycin or nitrogen starvation effectively induce autophagy and reduce global acetylome in *F. graminearum*. (a) GFP-Atg8 localization patterns in the wild type strain PH-1 grown in completed medium (CM). Bar=10 μm. Vacuoles were stained with the CMAC dye. (b) Mass spectrum data of rapamycin purified from S89 supernatant. The rapamycin produced model strain *S. hygroscopicus* NRRL5491 and pure rapamycin were used as positive control samples. (c) Autophagy flux of the fungus upon rapamycin or MM-N treatment. GFP-Atg8 labeled PH-1 was grown in CM before rapamycin or MM-N treatment. (d) Representative lysine acetylome profiling of *F. graminearum* upon nitrogen starvation. Total proteins were extracted from fresh mycelia and analyzed by immunoblot using an anti-acetyl-lysine antibody. Hybridization with an anti-GAPDH antibody served as an internal control. Asterisk indicates acetylated histones.

Figure S2

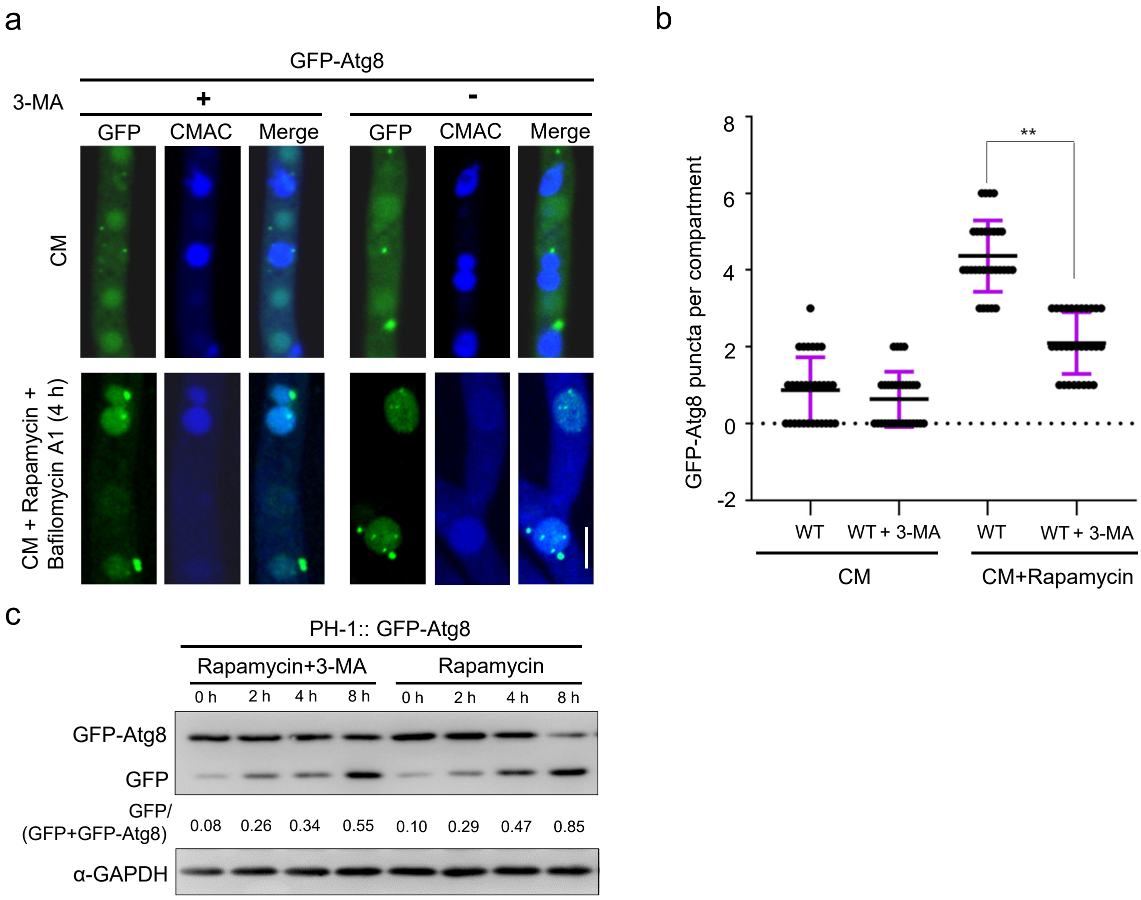

Fig. S2 3-MA treatment inhibits autophagy in *F. graminearum*. (a) GFP-Atg8 labelled PH-1 was grown in completed medium (CM) and treated with rapamycin in the presence/absence of 3-MA. Autophagic bodies in vacuoles were stabilized by the inhibitor bafilomycin A1. Vacuoles were stained with the CMAC dye. Bar=10 μm. (b) Quantification of the GFP-Atg8 puncta in (a). The data are presented as mean ± s. d., n = 30 compartments. **, $P < 0.01$. (c) Autophagy flux of the WT strain in the presence/absence of 3-MA upon rapamycin treatment.

Figure S3

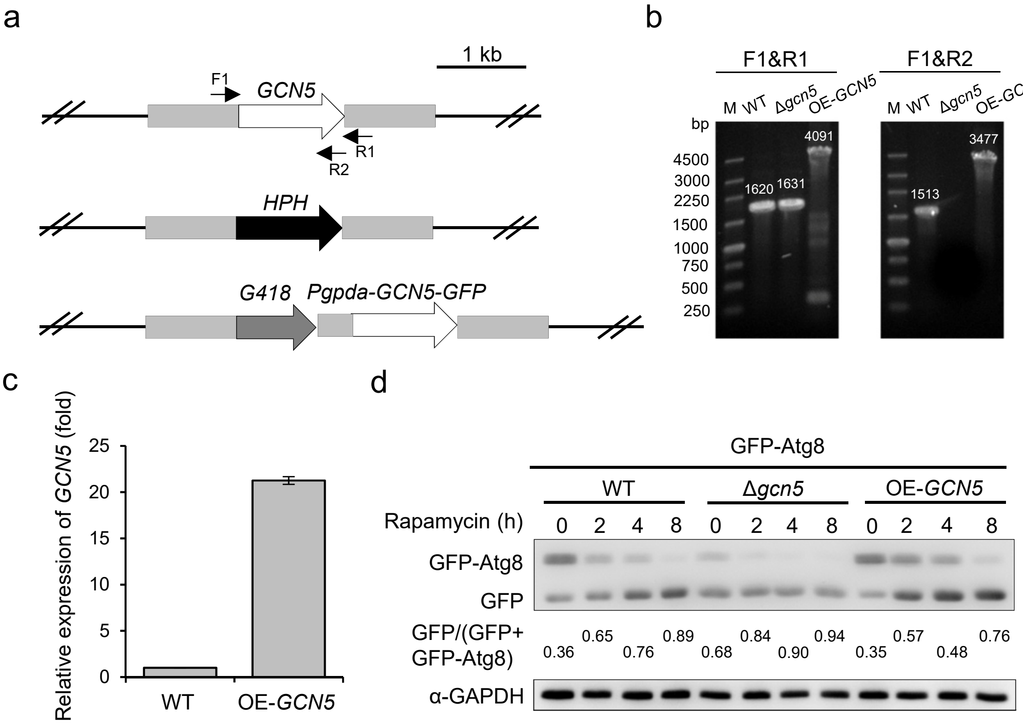

Fig. S3 Deletion and *in locus* overexpression of *GCN5*, and autophagy flux of the wild-type, Δ*gcn5* or OE-*GCN5* upon rapamycin treatment. (a) Diagram of *GCN5* deletion and *in locus* overexpression. F1 marked the forward primer for identification of genetic transformants of Gcn5. R1 and R2 are the designed reverse primers for identification. (b) Identification of *Δgcn5* and OE-*GCN5* strains by PCR assay. M: marker. (c) Relative mRNA level of *GCN5* in the wild-type PH-1 (WT) or OE-*GCN5* strain. Total RNA extracted from PH-1 or OE-*GCN5* was subjected to qRT-PCR assay. The expression of *ACTIN* in each sample was set as a reference. (d) Autophagy flux of the WT, Δ*gcn5* and OE-*GCN5* upon rapamycin treatment.

Figure S4

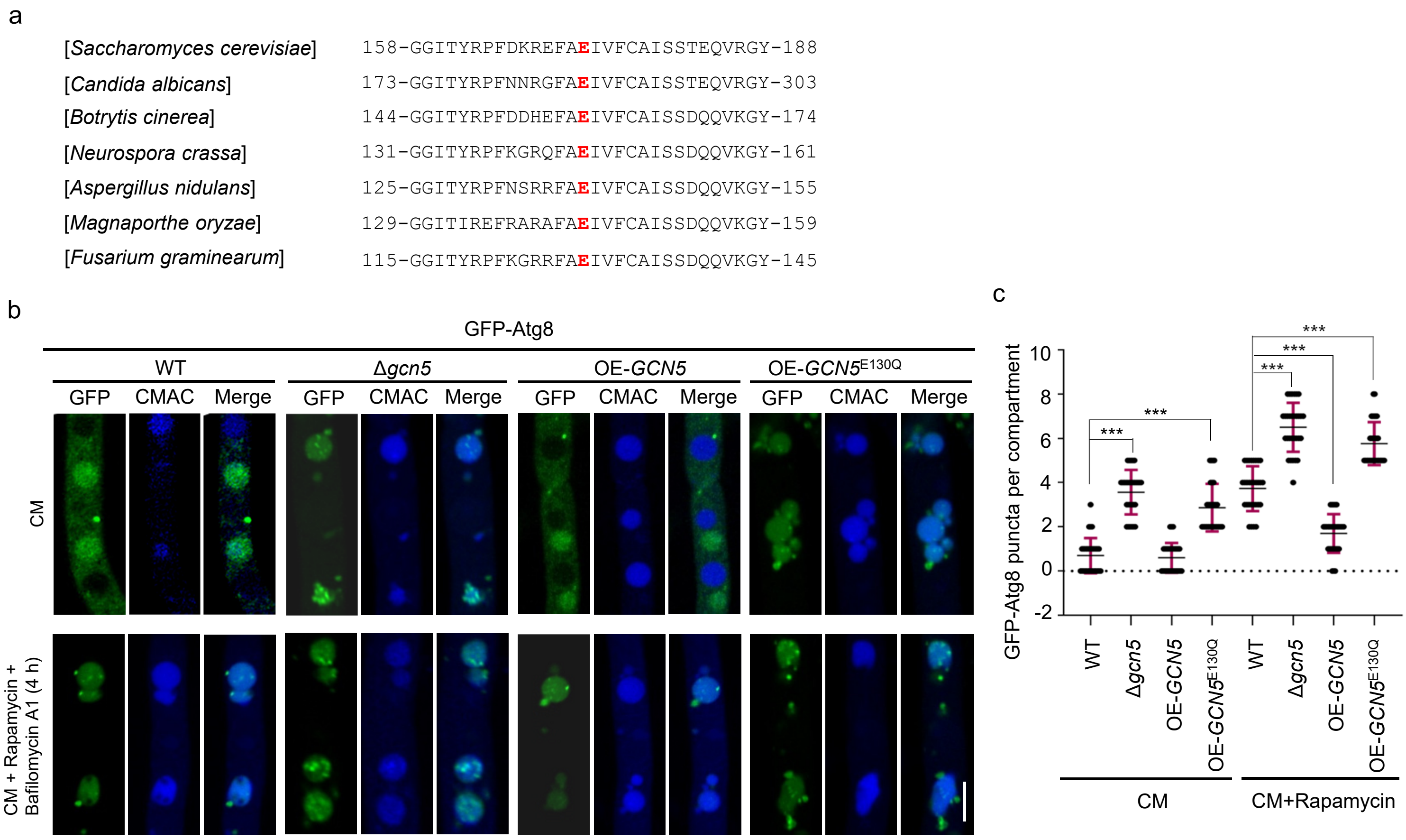

Fig. S4 The acetyltransferase activity of Gcn5 is critical for regulating autophagy. (a) Alignment of protein sequences of Gcn5 orthologs from various fungal species. The position highlighted in red indicates conserved 130th glutamic acid in *F. graminearum*. (b) GFP-Atg8 punctum formation in different strains during autophagy induced by rapamycin. Vacuoles were stained with the CMAC dye. Bar=10 μm. (c) Quantification of GFP-Atg8 punctum occurrence per compartment in (b). The data are presented as mean ± s. d., n = 30 compartments. **, *P* < 0.01.

Figure S5

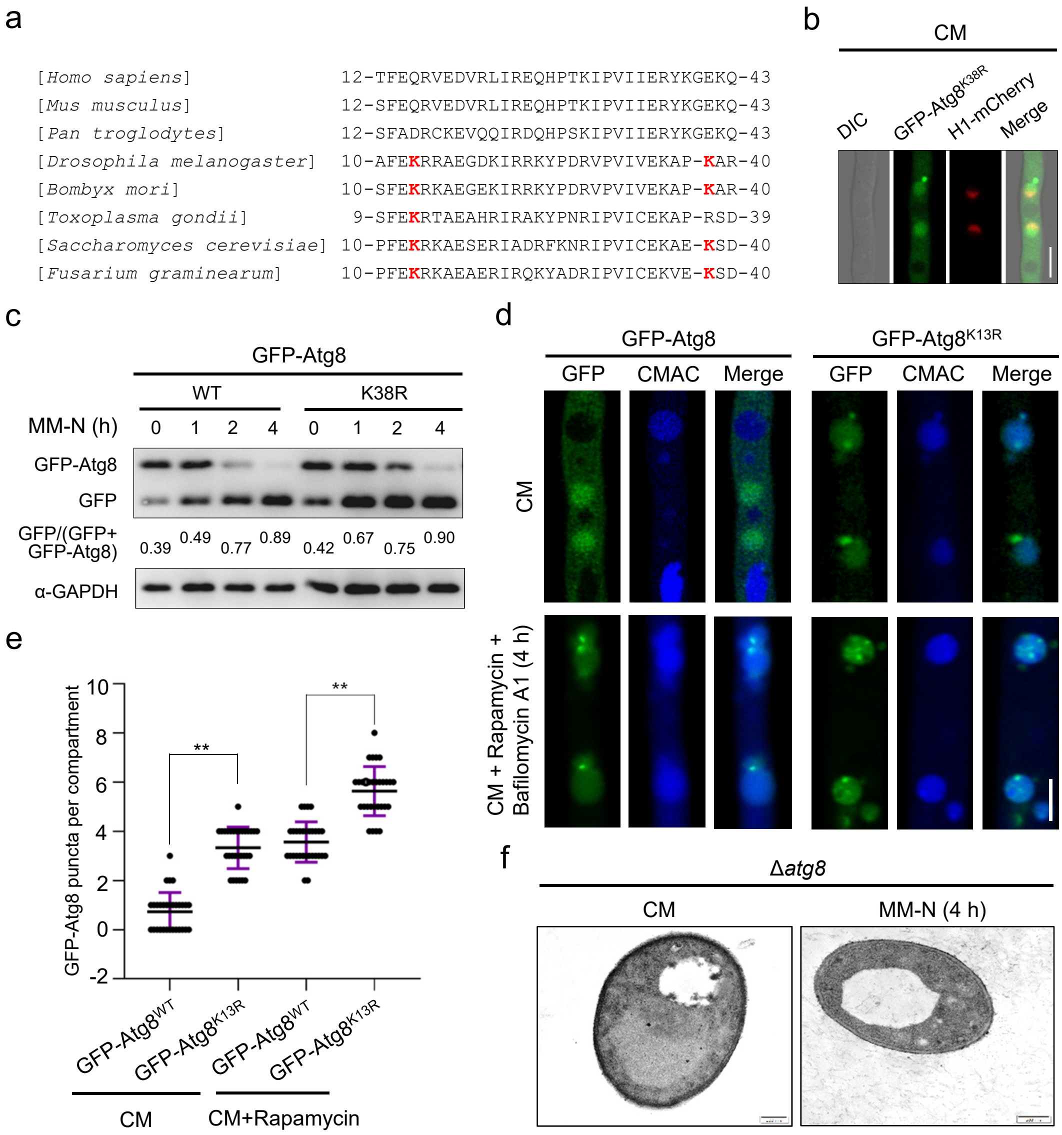

Fig. S5 K13 but not K38 acetylation in Atg8 is involved in autophagy. (a) Alignment of the protein sequences of Atg8 orthologs from various species. The positions highlighted in red indicates acetylated K13 and K38 lysine residues identified in Atg8 in *F. graminearum*. (b) Cellular localization of GFP-Atg8^{K38R}. Mycelia of GFP-Atg8^{K38R}-expressing strain were grown in CM. H1-mCherry was used as a nuclear marker. Bar=10 μm. (c) Immunoblot assay of the cleavage of GFP-Atg8^{K38R} or GFP-Atg8 upon nitrogen starvation. (d) GFP-Atg8 punctum formation in different strains during autophagy induced by rapamycin. Vacuoles were stained with the CMAC dye. Bar=10 μm. (e) Quantification of GFP-Atg8 punctum occurrence per compartment in (d). The data are presented as mean ± s. d., n = 30 compartments. **, $P < 0.01$. (f) TEM images of the autophagic structures in Δ*atg8* mutant under nitrogen starvation.

Figure S6

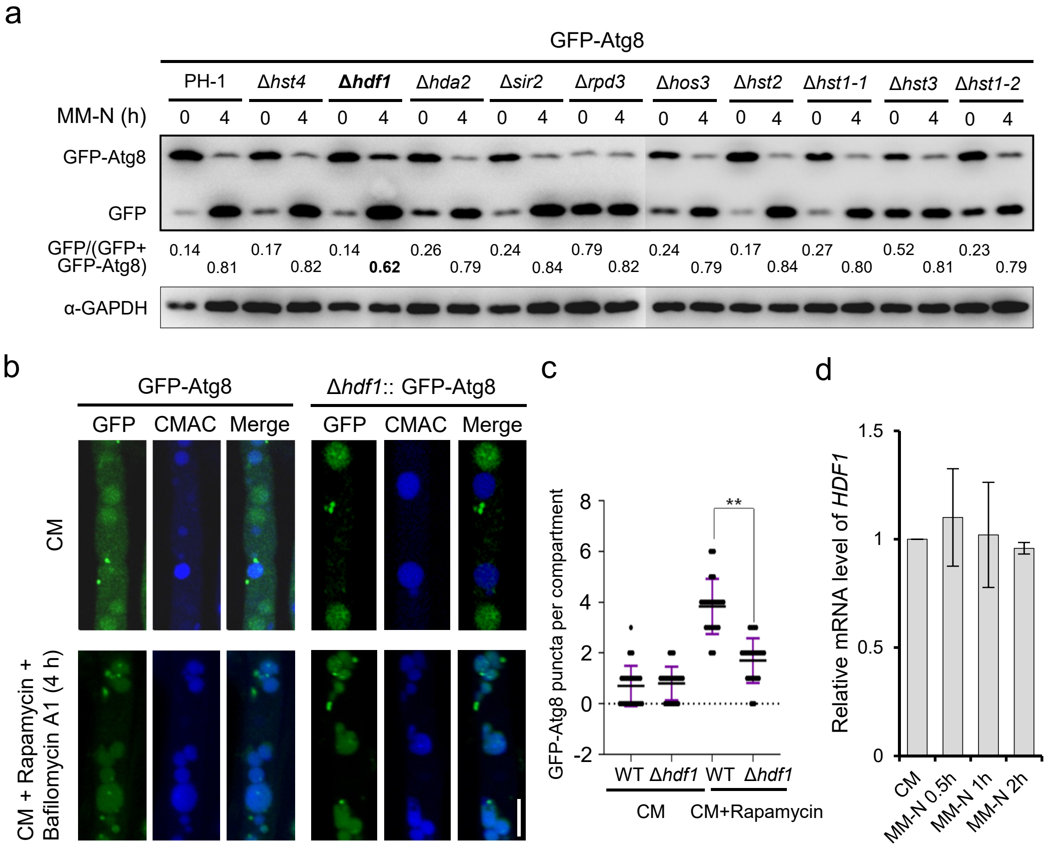

Fig. S6 Deacetylase Hdf1 plays a positive role in autophagy regulation. (a) Autophagy flux of various deletion mutants of deacetylases in *F. graminearum*. Total proteins extracted from the mycelia of various mutants grown in CM (indicated as 0 h) or MM-N for 4 h were analyzed by immunoblot assays with the anti-GFP antibody. The extent of autophagy was estimated and indicated underneath the blot. The intensities of bands were quantified with ImageJ. Hybridization with the α-GAPDH antibody served as an internal control. (b) GFP-Atg8 punctum formation in wild type PH-1 and Δ*hdf1* during autophagy induced by rapamycin. Vacuoles were stained with the CMAC dye. Bar=10 μm. (c) Quantification of GFP-Atg8 punctum occurrence per compartment in (b). The data are presented as mean ± s. d., n = 30 compartments. **, $P < 0.01$. (d) Relative mRNA level of *HDF1* in CM or MM-N at the indicated time points. Total RNA was extracted from the mycelia and subjected to a qRT-PCR assay. The expression of *ACTIN* in each sample was used as a reference.

Figure S7

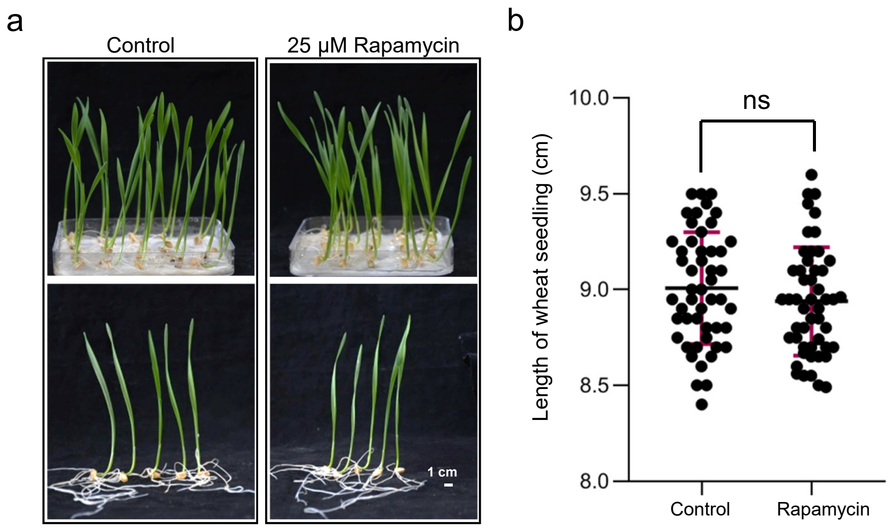

Fig. S7 Wheat plants are insensitive to rapamycin. (a) Representative wheat seedlings on the 4th day post-rapamycin treatment. Germinated wheat seeds were grown in a light-dark (12/12) growth chamber after rapamycin (25 μM) treatment. Diluted DMSO was used as non-treatment control. Bar=1 cm. (b) Quantification of the length of seedlings in (a). The data are presented as mean ± s. d., n =50.

Figure S8

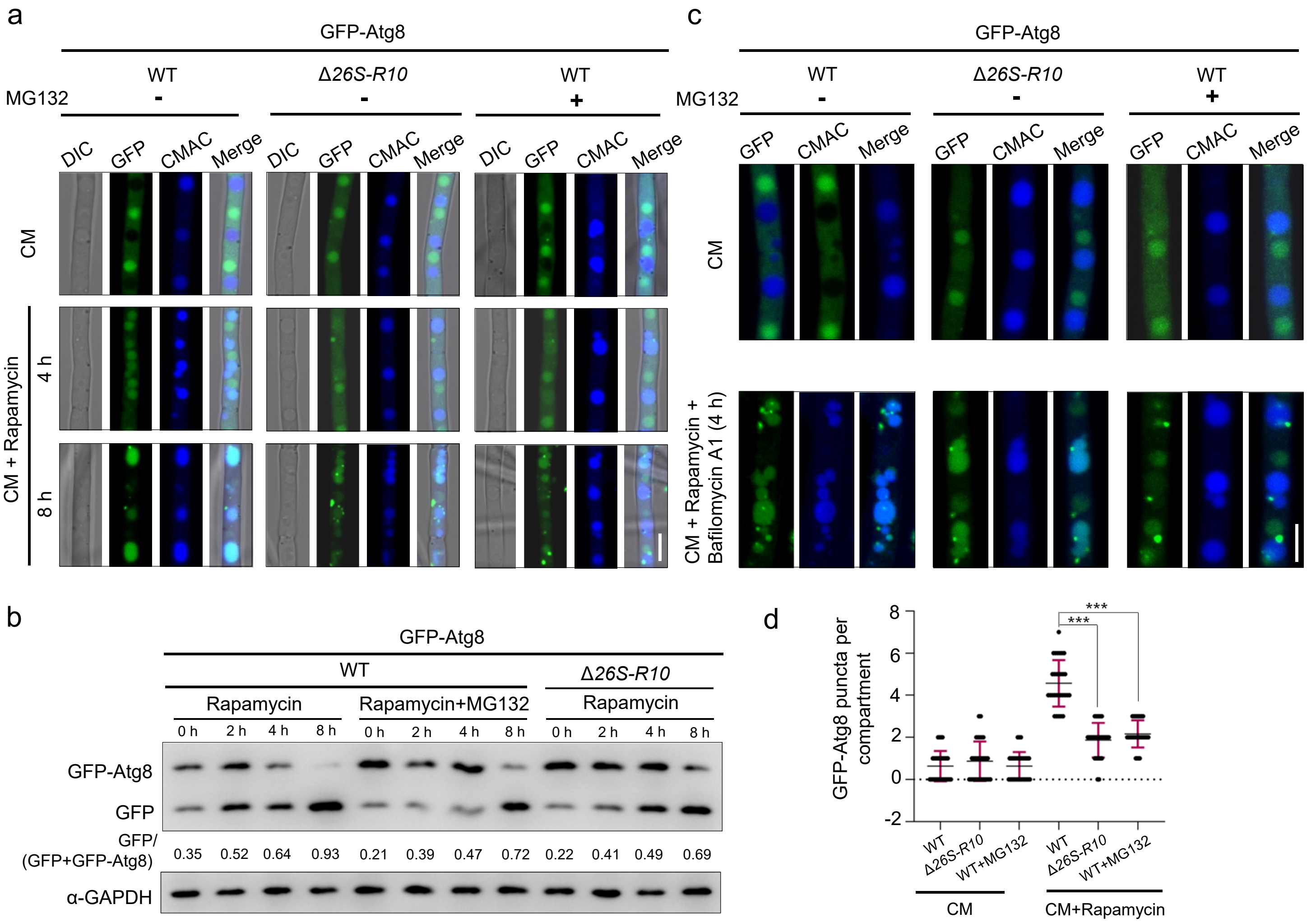

Fig. S8 Inhibition of ubiquitin-proteasome system suppresses autophagy flux induced by rapamycin in *F. graminearum*. (a) GFP-Atg8 translocation during autophagy induced by rapamycin in the wild type PH-1 in the presence/absence proteasome inhibitor MG132, and 26S proteasome defective mutant *Δ26S-RS10* in *F. graminearum*. Vacuoles were stained with the CMAC dye. Bar=10 μm. (b) Autophagy flux detected by GFP cleavage assay in different strains or treatments. (c) GFP-Atg8 punctum formation in wild type PH-1, *Δ26S-RS10* or MG132 treated PH-1 during autophagy induced by rapamycin. Bar=10 μm. (d) Quantification of GFP-Atg8 punctum occurrence per compartment in (c). The data are presented as mean ± s. d., n = 30 compartments. **, *P* < 0.01.

Figure S9

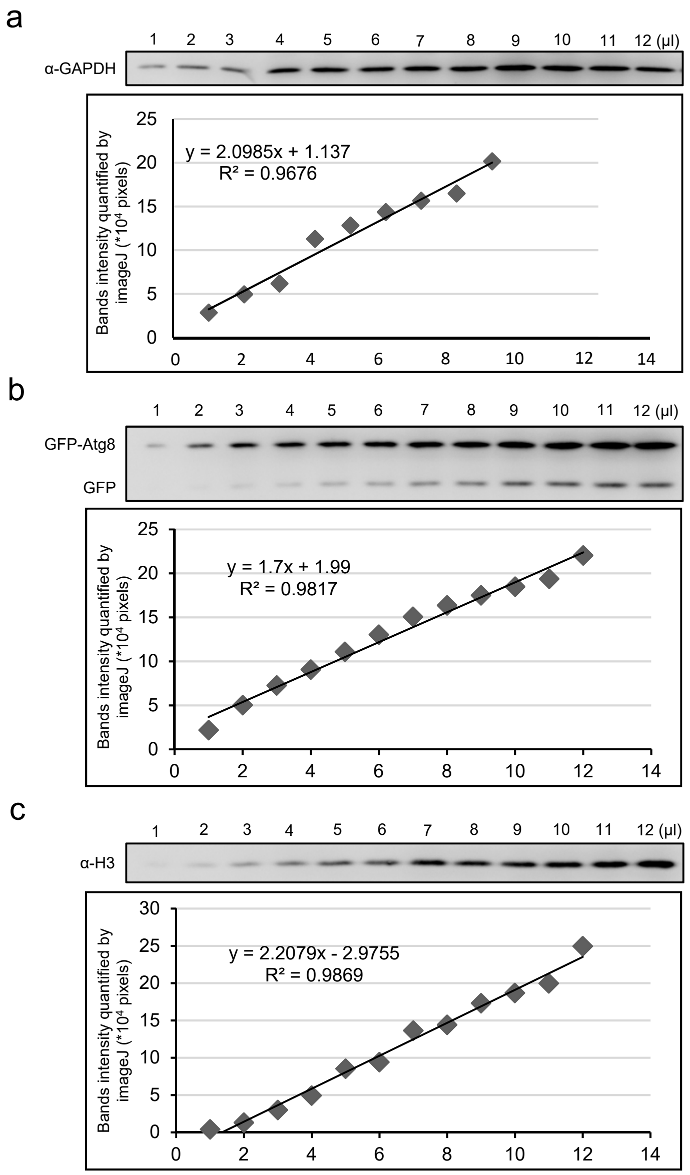

Fig. S9 Linear range of western blot bands. Total protein lysates of the wild type strain labeled with GFP-Atg8 were quantified with a BCA protein assay kit, and then subjected to western blotting in a volume range from 1 to 12 µL. The protein GAPDH (a, b), GFP-Atg8 (c, d), and H3 (e, f) were detected with corresponding antibodies, the intensities of specific bands were quantified with ImageJ and analyzed with linear regression.
